# Pyruvate kinase M1 regulates butyrate metabolism in cancerous colonocytes

**DOI:** 10.1038/s41598-022-12827-9

**Published:** 2022-05-24

**Authors:** Bohye Park, Ji Yeon Kim, Olivia F. Riffey, Presley Dowker-Key, Antje Bruckbauer, James McLoughlin, Ahmed Bettaieb, Dallas R. Donohoe

**Affiliations:** 1 Department of Nutrition, University of Tennessee, 1215 W. Cumberland Ave., Knoxville, TN 37996 USA; 2 Department of Microbiology, University of Tennessee, Knoxville, TN 37996 USA; 3 Graduate School of Genome Science and Technology, University of Tennessee, Knoxville, TN 37996 USA; 4 Department of Biochemistry, Cellular and Molecular Biology, University of Tennessee, Knoxville, TN 37996 USA; 5 University of Tennessee Medical Center Biorepository, Knoxville, TN 37920 USA

**Keywords:** Biochemistry, Biological techniques, Cancer, Cell biology, Molecular biology, Physiology, Diseases, Gastroenterology

## Abstract

Colorectal cancer (CRC) cells shift metabolism toward aerobic glycolysis and away from using oxidative substrates such as butyrate. Pyruvate kinase M1/2 (PKM) is an enzyme that catalyzes the last step in glycolysis, which converts phosphoenolpyruvate to pyruvate. M1 and M2 are alternatively spliced isoforms of the *Pkm* gene. The PKM1 isoform promotes oxidative metabolism, whereas PKM2 enhances aerobic glycolysis. We hypothesize that the PKM isoforms are involved in the shift away from butyrate oxidation towards glycolysis in CRC cells. Here, we find that PKM2 is increased and PKM1 is decreased in human colorectal carcinomas as compared to non-cancerous tissue. To test whether PKM1/2 alter colonocyte metabolism, we created a knockdown of PKM2 and PKM1 in CRC cells to analyze how butyrate oxidation and glycolysis would be impacted. We report that butyrate oxidation in CRC cells is regulated by PKM1 levels, not PKM2. Decreased butyrate oxidation observed through knockdown of PKM1 and PKM2 is rescued through re-addition of PKM1. Diminished PKM1 lowered mitochondrial basal respiration and decreased mitochondrial spare capacity. We demonstrate that PKM1 suppresses glycolysis and inhibits hypoxia-inducible factor-1 alpha. These data suggest that reduced PKM1 is, in part, responsible for increased glycolysis and diminished butyrate oxidation in CRC cells.

## Introduction

Colorectal cancer (CRC) is the second leading cause of cancer deaths in the United States^1^. Cancer cells primarily undergo rapid glycolysis instead of oxidative metabolism, and this metabolic shift is associated with several critical genetic modifications^2^. Specifically, colorectal cancer cells increase glycolysis and decrease the oxidation of butyrate^3,4^. Butyrate is a short-chain fatty acid (SCFA) that is a bacterial-derived product from the fermentation of dietary fiber^5^. Normal colonocytes use butyrate as their primary energy source^6,7^. In contrast, cancerous colonocytes prefer to utilize glucose, and there is a concomitant diminishment in butyrate oxidation^4,8^. The metabolic shift toward increased glucose utilization is caused by an upregulation of enzymes that promote glycolysis in CRC^9^. Therefore, it is important to understand the mechanisms that contribute to the metabolic shift away from butyrate oxidation toward glycolysis in the cancerous colonocyte.

Much effort has been put forth to identify and characterize the genetic and environmental factors that influence the development of CRC. The isoform of the metabolic enzyme pyruvate kinase called PKM2 has been repeatedly identified in clinical studies as being upregulated in colorectal cancer biopsies^10–15^. PKM2 is one of four isoforms (PKL, PKR, and PKM1 are the others) that catalyze the final step in glycolysis, where phosphoenolpyruvate is converted to pyruvate, and ATP is formed^16^. Importantly, each isoform has different enzyme kinetics and a distinct function. PKM2 and PKM1 are both expressed in the colon, however, PKM1 is generally associated with differentiated colonocytes to promote oxidative metabolism and PKM2 is associated with proliferating and colorectal cancer cells to enhance aerobic glycolysis^17,18^. Studies have found that elevated PKM2 expression in human cancers, including CRC, is associated with a poor prognosis and outcome in patients^19^. This increase in PKM2 is so apparent that PKM2 has even been coined as a potential CRC biomarker^12,14,19^. Nevertheless, the importance of PKM2 in the development and progression of CRC is unclear. However, given its place in the glycolytic pathway, a metabolic role seems plausible.

In this study, experiments were conducted to distinguish the role of two different pyruvate kinase isoforms in CRC cell metabolism. There was a particular emphasis toward characterizing the mechanism by which PKM1/2 alters butyrate metabolism in the cancerous colonocyte. Similar to other studies, we report that PKM2 is upregulated in CRC patient biopsies compared to the normal colon tissues. In addition, we also find that PKM1 expression was significantly diminished in CRC samples, suggesting a potential role of this isoform. Towards this end, we show that PKM1, rather than PKM2, regulates butyrate oxidation in CRC cells. We further demonstrate that diminished PKM1 causes the cell to shunt metabolism toward glycolysis away from butyrate oxidation. This shift in metabolism is mediated through elevated hypoxia-inducible factor 1 alpha (HIF1α), a transcription factor that promotes glycolysis. Moreover, short-chain acyl dehydrogenase (SCAD), a necessary enzyme in the oxidation of butyrate, decreased as HIF1α increased in CRC cells. Therefore, diminished PKM1 expression along with upregulated HIF1α and downregulated SCAD is a key mechanism responsible for the metabolic shift in CRC cells.

## Results

### PKM2 expression is increased in colorectal cancer

**Figure 1:**
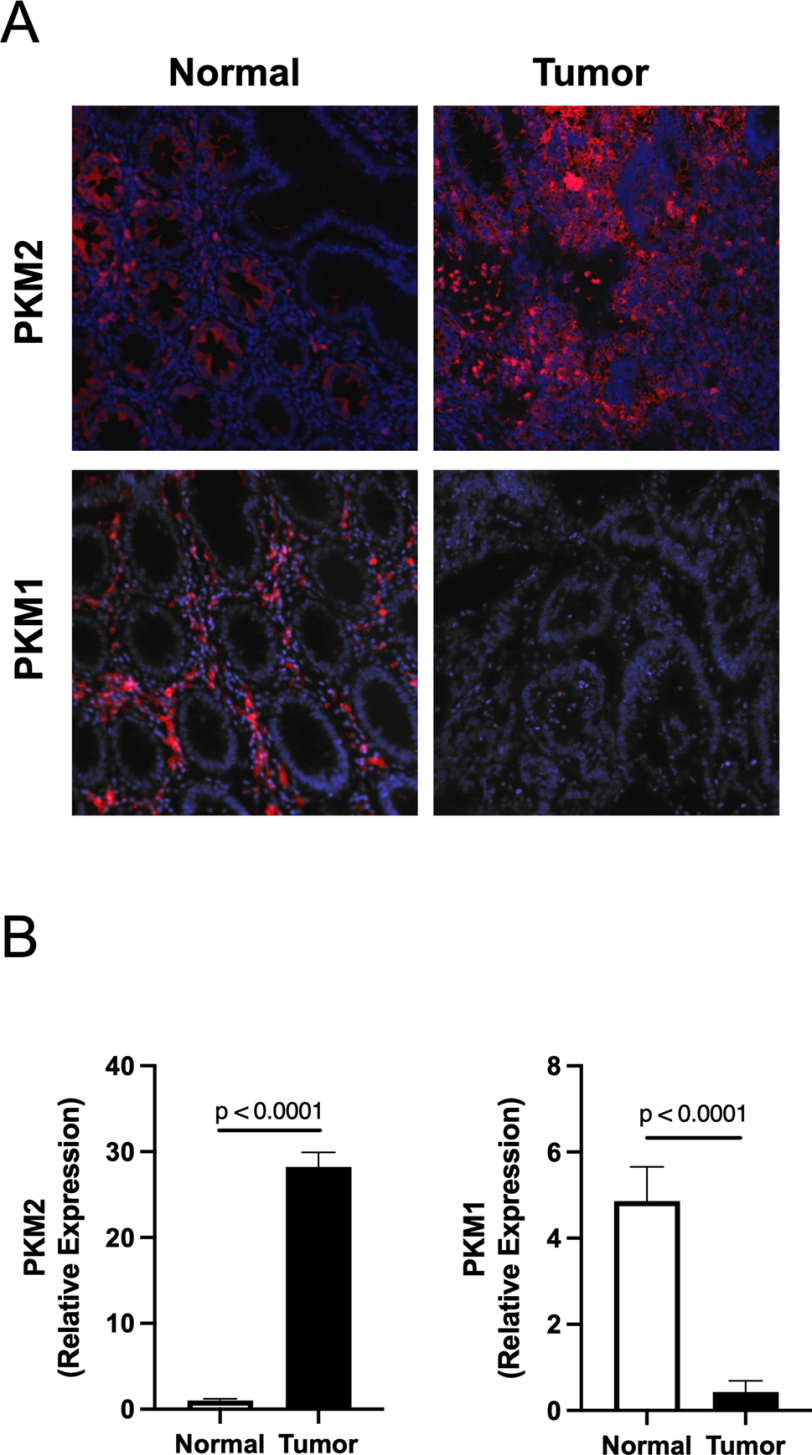
Expression of PKM isoform in human non-cancerous and cancerous colorectal tissues. (**A**) Immunofluorescence staining for PKM2 and PKM1 expression on non-cancerous colorectal tissue and colorectal cancer tissue. (**B**) Quantification of immunofluorescence data with relative expression of PKM2 and PKM1 in each group. Error bars are mean ± SEM for ten independent sections.

Several groups have reported that PKM2 is elevated in colorectal cancer samples^10,17,19^. However, it is unclear whether this increase in PKM2 plays any role in disease progression or outcome. In collaboration with the University of Tennessee Medical Center Biobank, we obtained clinical samples of colorectal cancer tissue and adjacent non-cancerous colon tissue. We performed immunofluorescence staining on these samples to compare the different PKM isoforms in cancerous and non-cancerous colorectal tissue. PKM2 expression was found to be increased in colorectal carcinomas as opposed to PKM1, which was decreased when compared to adjacent non-cancerous tissue (Fig. 1A). In CRC tissues, PKM1 was barely detectable and showed a significant diminishment in colorectal carcinomas (Fig. 1B). These data suggest that the PKM isoforms are differentially expressed in colorectal cancer and may allude to the functional or metabolic importance of these proteins in the disease.

### Targeting of PKM2 in colorectal cancer cells and its role in butyrate oxidation

**Figure 2:**
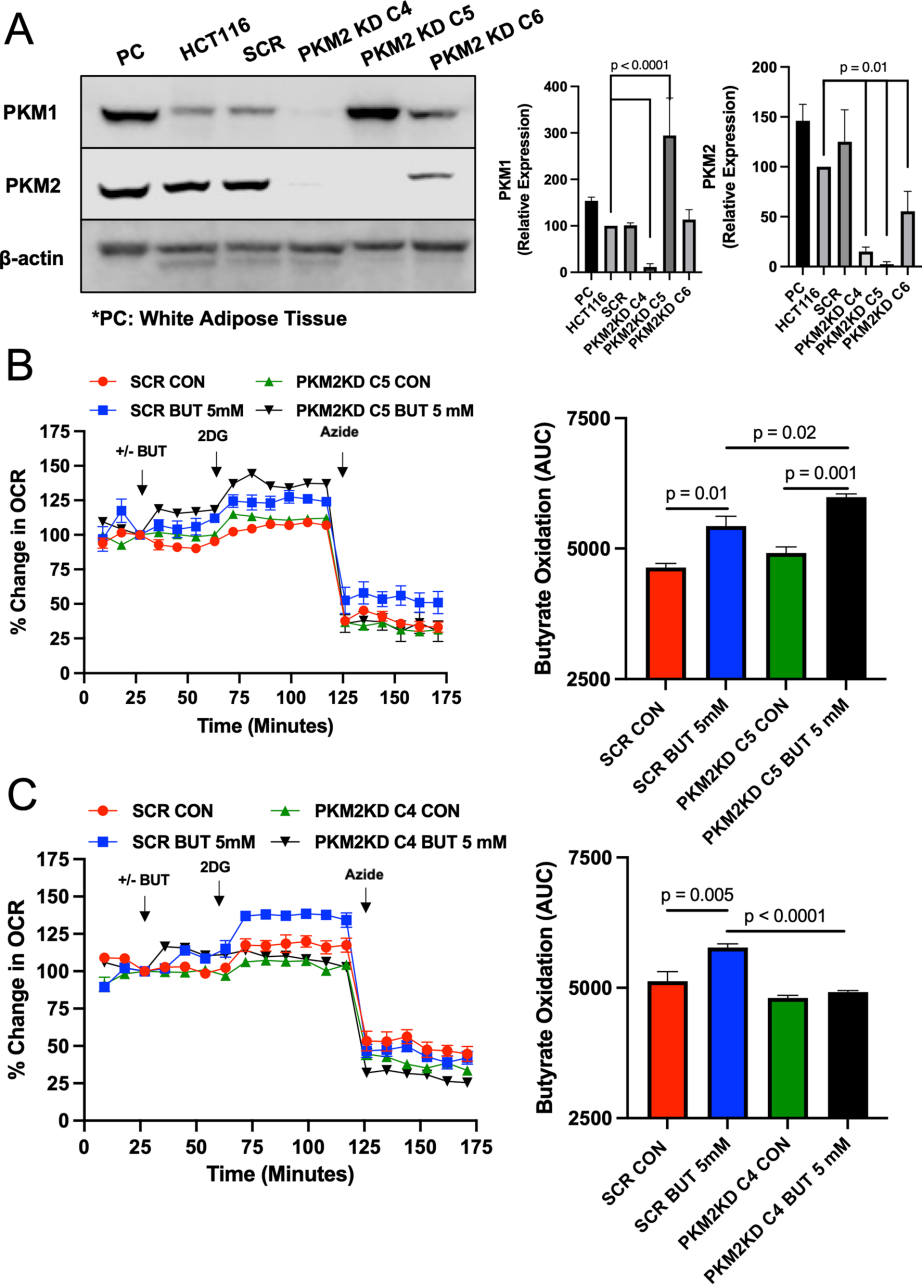
Butyrate oxidation in PKM2 knockdown HCT116 colorectal cancer cells. (**A**) Western blot analysis of PKM1 and PKM2 expression in the positive control (PC; Mouse White Adipose Tissue), HCT116, scramble (SCR), and different PKM1/2 targeted knockdown cells with β-actin as a loading control. Quantification of the western blot is shown in the right panel. For statistical analysis, western blot was conducted 5 times. Error bars are mean ± SEM. (**B**) Percent change in oxygen consumption rate (OCR) relative to baseline in which SCR and PKM2 KD C5 cells treated with and without butyrate (5 mM). Total contribution of butyrate toward OCR (%) is observed after injection of 2-deoxyglucose (2DG). The right panel shows the area under the curve (AUC) analysis from OCR measurements taken after 2DG injection but before azide injection. These measurements represent butyrate oxidation (arbitrary units). (**C**) Percent change in OCR relative to baseline in which SCR and PKM2 KD C4 cells treated with and without butyrate (5 mM). The right panel shows AUC analysis from OCR measurements taken after 2DG injection but before azide injection. These measurements represent butyrate oxidation (arbitrary units). Data points represent the average OCR (%) over 3–5 replicates per condition for butyrate oxidation measurements. Error bars are mean ± SEM.

To study the role of PKM2 in colorectal cancer cells, we targeted PKM2 with shRNA and attempted to generate a stable knockdown cell line. Derived from the parental HCT116 colon carcinoma cell line, we isolated three clones (C4, C5, and C6) and analyzed the knockdown of PKM2 by western blot. As shown in Fig. 2A, PKM2 was barely detected in clone 4 (C4) and clone 5 (C5) and was diminished in clone 6 (C6) compared to the parental HCT116 and scrambled mock cell lines. In addition, we found that PKM1 expression in the clones differed (Fig. 2A). This was most apparent in C4, where PKM1 expression was almost abolished, and C5, which showed overexpression of PKM1 compared to scrambled and HCT116 cells. We are not sure how or why this happened; however, these clones provide us an opportunity to study the role of PKM1 and PKM2 in colorectal cancer cell metabolism. So, we checked the expression of Polypyrimidine tract-binding protein 1 (PTBP1), which is one of the PKM splicer genes promoting PKM2 expression^20–23^. Dysregulation of microRNA (miRNA) causes increased PTBP1 and PKM2 during carcinogenesis^24^. In addition, PTBP1 is negatively regulated by tissue-specific miRNA^25^. Specifically, reduced miRNA, MIR1-3p, MIR124-3p, and MIR133b, has been observed in CRC^25–28^. In different PKM2 knocked down cones, we find that the level of PTBP1 is decreased in scrambled and C4, whereas PTBP1 in C5 is significantly increased (Supplemental Fig. 1). Furthermore, we verified whether the knockdown or expression patterns of PKM1/PKM2 are maintained in each clone through several passages (Supplemental Fig. 2). No experiments used the generated cell lines past passage 3.

Since bacterial-derived butyrate is an energetic substrate for colonocytes, and cancerous colonocytes have been reported to show diminished oxidation of butyrate, we decided to examine changes in butyrate oxidation in C4 and C5. The Seahorse XFe24 Extracellular Flux Analyzer, which is an instrument that measures oxidative (oxygen consumption) and non-oxidative (glycolysis or lactate production) metabolism in cells was utilized to analyze butyrate oxidation in actively respiring cells^3,29,30^. Data acquired with this instrument showed that butyrate oxidation was enhanced in C5 cells as compared to the cell line expressing the scrambled mock shRNA (Fig. 2B). This was in line with diminished PKM2 expression leading to an increase in butyrate oxidation. However, when we tested C4, we found that butyrate oxidation was significantly diminished in this clone compared to the scramble control cells (Fig. 2C). Importantly, C4 cells were able to oxidize butyrate, however, to a lower level than scrambled cells. Subsequently, C4 and C5 both have reduced PKM2, to a similar extent, therefore these data suggest that PKM2 diminishment is not the key factor in regulating the oxidation of butyrate. Instead, PKM1, which was overexpressed in C5, and almost abolished in C4, was the more likely factor in explaining these opposing results.

### Rescue with PKM1 in C4 restores butyrate oxidation

**Figure 3:**
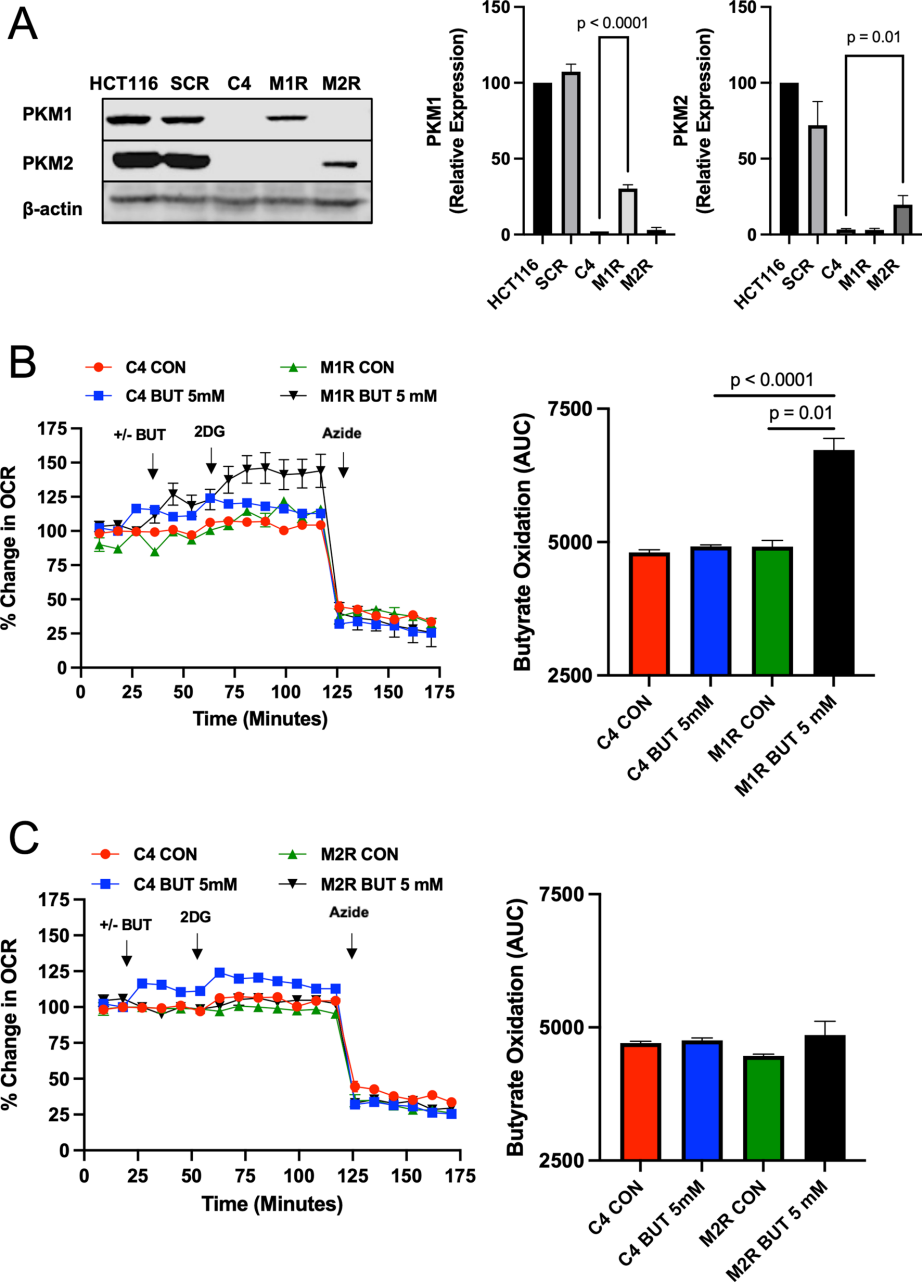
Butyrate oxidation is PKM1 dependent rather than PKM2 in HCT116 colorectal cancer cells. (**A**) Western blot analysis of PKM1 and PKM2 expressions in HCT116, SCR, and re-expressed PKM1 (M1R) and PKM2 (M2R) cells with β-actin as a loading control. Quantification of the western blot is shown in the right panel. For statistical analysis, western blot was conducted 5 times. (**B**) Percent change in oxygen consumption rate (OCR) relative to baseline in which C4 and M1R cells treated with and without butyrate (5 mM). Total contribution of butyrate toward OCR (%) is observed after injection of 2-deoxyglucose (2DG). The right panel shows the area under the curve (AUC) analysis from OCR measurements taken after 2DG injection but before azide injection. These measurements represent butyrate oxidation (arbitrary units). (**C**) Percent change in OCR relative to baseline in which C4 and M2R cells treated with and without butyrate (5 mM). The right panel shows the area under the curve analysis from OCR measurements taken after 2DG injection but before azide injection. These measurements represent butyrate oxidation (arbitrary units). Data points represent the average OCR (%) over 3–5 replicates per condition for butyrate oxidation measurements. Error bars are mean ± SEM.

To test whether PKM1 is the factor regulating differential butyrate oxidation in these colorectal cancer cells, we re-expressed PKM1 in C4 cells to observe whether this rescued the oxidation of butyrate to a level comparable to scrambled control cells. In addition, we re-expressed only PKM2 in C4 cells to analyze how that would affect butyrate oxidation. In the C4 cells, PKM1 (M1R) or PKM2 (M2R) protein was increased as compared to C4 non-rescue cells (Fig. 3A). In the M1 rescue (M1R) cells, we found that the oxidation of butyrate was significantly increased compared to C4 non-rescue cells **(**Fig. 3B). However, expressing PKM2 in C4 cells (M2R) showed no change in butyrate oxidation compared to C4 non-rescue cells **(**Fig. 3C). Taken together, these data suggested that PKM1 is largely responsible for regulating butyrate oxidation. However, these data do not preclude a role for PKM2 in the process, but rather point to PKM1 as having the biggest impact on butyrate oxidation in these CRC cells. Furthermore, we confirmed the role of PKM1 in butyrate oxidation using the HEK293 (human embryonic kidney) cell line, which expresses PKM2, but not PKM1^31^. To test whether PKM1 is the major factor regulating butyrate oxidation, we transfected PKM1 (M1R) in the HEK293 cell line, and PKM1 was stably expressed in clone 2 (C2) cells (Supplemental Fig. 3A). In the C2 HEK293 cell line that expressed PKM1, we found that the butyrate oxidation was significantly increased compared to scramble HEK293 cells (Supplemental Fig. 3B). These data further support the role of PKM1 promoting butyrate oxidation, and show that PKM1 also has this effect in a non-cancerous or colorectal cell line.

**Figure 4:**
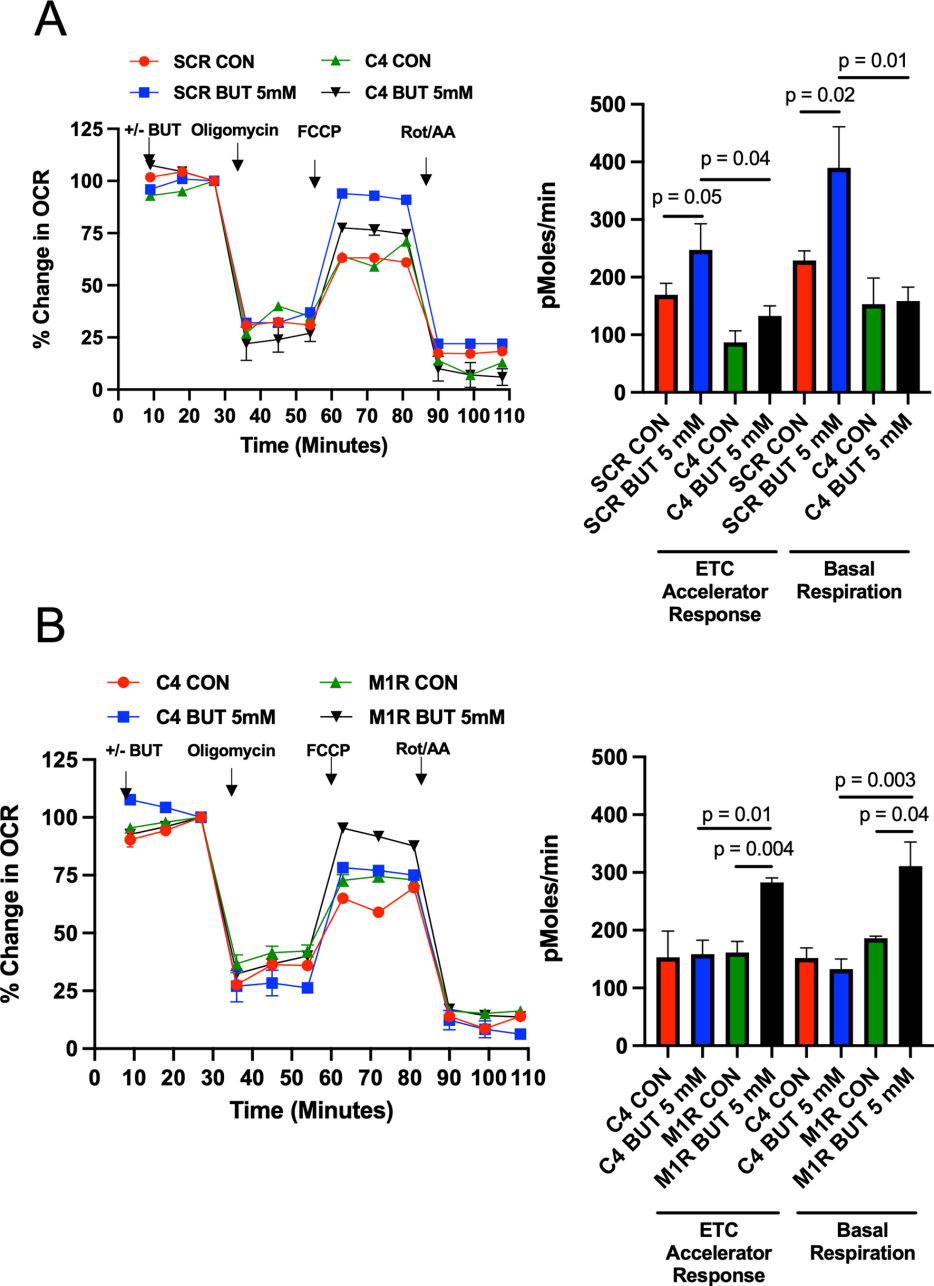
PKM1 is responsible for mitochondrial function in HCT116 colorectal cancer cells. (**A**) Percent change in oxygen consumption rate (OCR) relative to baseline in which SCR and C4 cells respond to oligomycin, FCCP, and Antimycin A/Rotenone. The right panel shows the calculated electron transport chain (ETC) accelerator response and basal respiration. (**B**) Percent change in OCR relative to baseline in which C4 and M1R cells respond to oligomycin, FCCP, and Antimycin A/Rotenone. The right panel shows the calculated ETC accelerator response and basal respiration. For these experiments, each cell line was treated with or without butyrate (5 mM). These measurements represent the mitochondrial function. Each data point represents the average OCR (%) over 3–5 replicates per condition. Error bars are mean ± SEM.

To test whether mitochondrial function, in regard to response to butyrate, is altered in C4 cells, we used oligomycin and FCCP to measure how scrambled control and C4 cells would respond. Oligomycin is an inhibitor of ATP synthase that reduces ATP-linked oxygen consumption, while FCCP is an uncoupling agent used to show the maximal respiratory capacity. After injection of FCCP, butyrate caused an increased electron transport chain (ETC) accelerator response in scramble control cells (Fig. 4A). However, C4 cells, which show a large diminishment in PKM2 and PKM1, did not show this same response. The C4 cells also showed no response to butyrate in regard to basal respiration. This diminishment in ETC accelerator response and basal respiration was rescued by re-addition of PKM1 in M1R cells (Fig. 4B). Also, to test whether PKM2 re-addition affects mitochondrial function response to butyrate, we compared C4 cells and M2R cells. Here, we found that the diminishment in ETC accelerator response and basal respiration in C4 was not rescued by re-addition of PKM2 in M2R cells (Supplemental Fig. 4). Thus, these data show that PKM1 is responsible for the differences in mitochondrial function and an overall diminishment in basal respiration in C4 cells as compared to scrambled control cells that are rescued by PKM1 re-expression.

### Molecular consequences of diminished PKM1 and PKM2

**Figure 5:**
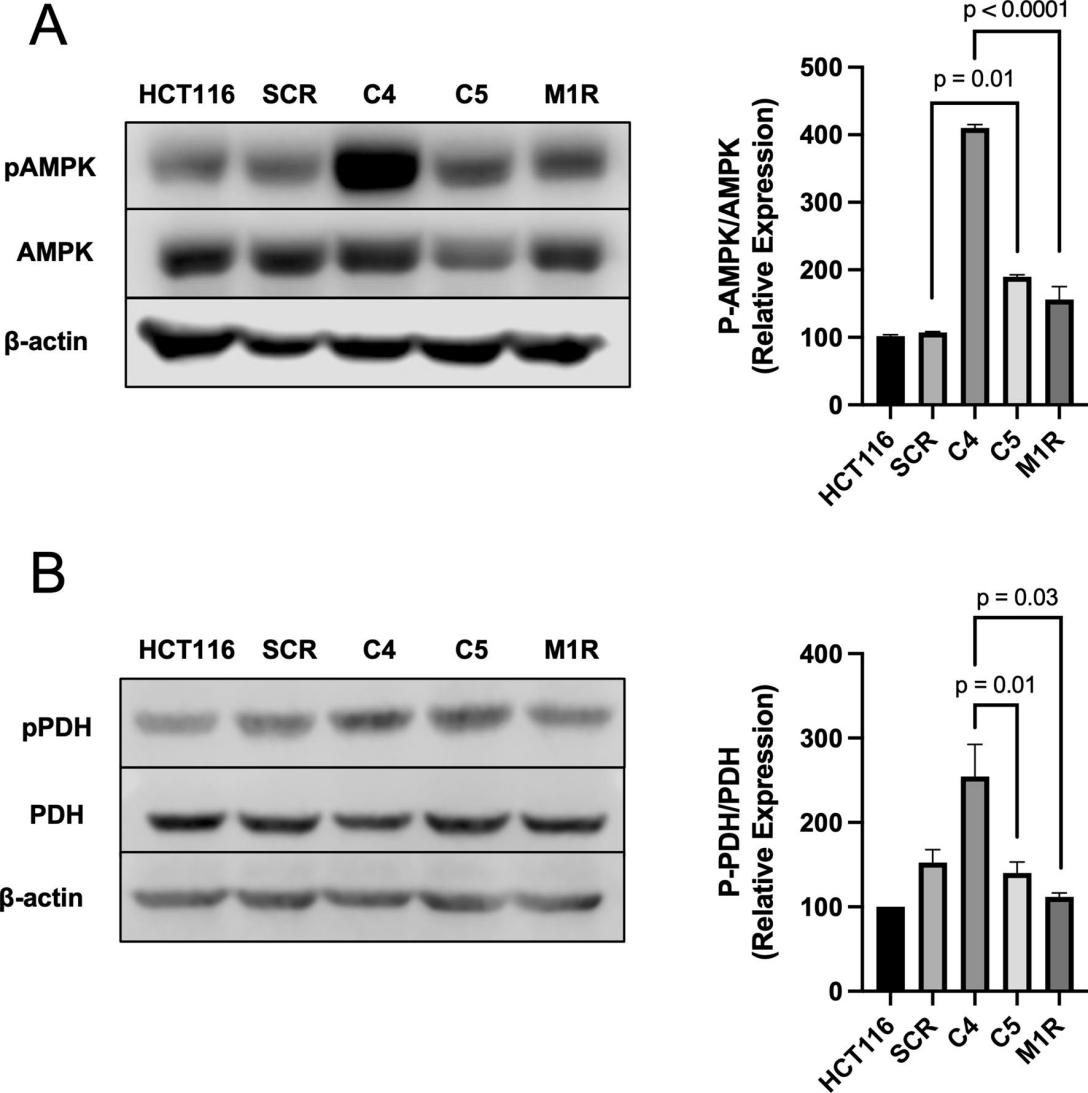
Molecular consequences in PKM1- and PKM2-diminished HCT116 colorectal cancer cells. (**A**) Western blot analysis of phospho-AMPK (Thr172) and total AMPK levels in HCT116, SCR, C4, C5, and M1R cells with β-actin as a loading control. The right panel shows the quantification of p-AMPK levels relative to total AMPK levels. (**B**) Western blot analysis of phospho-PDH (Ser293) and total PDH levels in HCT116, SCR, C4, C5, and M1R cells with β-actin as loading controls. The right panel shows the quantification of p-PDH levels relative to total PDH levels. For statistical analysis, western blot was conducted 5 times. Error bars are mean ± SEM.

Since C4 cells had decreased basal respiration, we next sought to test whether they also showed an increase in the phosphorylation of AMPK (Thr172) as a proxy of energetic stress. As expected, C4 cells showed an elevation in phospho-AMPK compared to parental HCT116 and scrambled control cells (Fig. 5A). Re-expression of PKM1 in M1R cells diminished this activation of phospho-AMPK. Despite this activation of AMPK, one of many downstream targets, ACC, did not show elevated phosphorylation (Supplemental Fig. 5).

C4 cells that also showed a significant diminishment in PKM1 and PKM2 displayed a decrease in oxidation of butyrate, which may result in the cells shunting metabolism toward glycolysis. In such a case, the pyruvate dehydrogenase complex (PDH) becomes inactivated through the phosphorylation of the E1 complex member. Towards this end, C4 cells exhibited an increase in phospho-PDH as compared to parental HCT116 cells and scrambled control cells. However, this increase in phospho-PDH was blocked in M1R cells (Fig. 5B). This suggested that the loss of PKM1 decreases oxidative metabolism of glucose and promotes glycolysis.

### Loss of PKM1 induces HIF-1alpha that decreases short-chain acyl dehydrogenase

**Figure 6:**
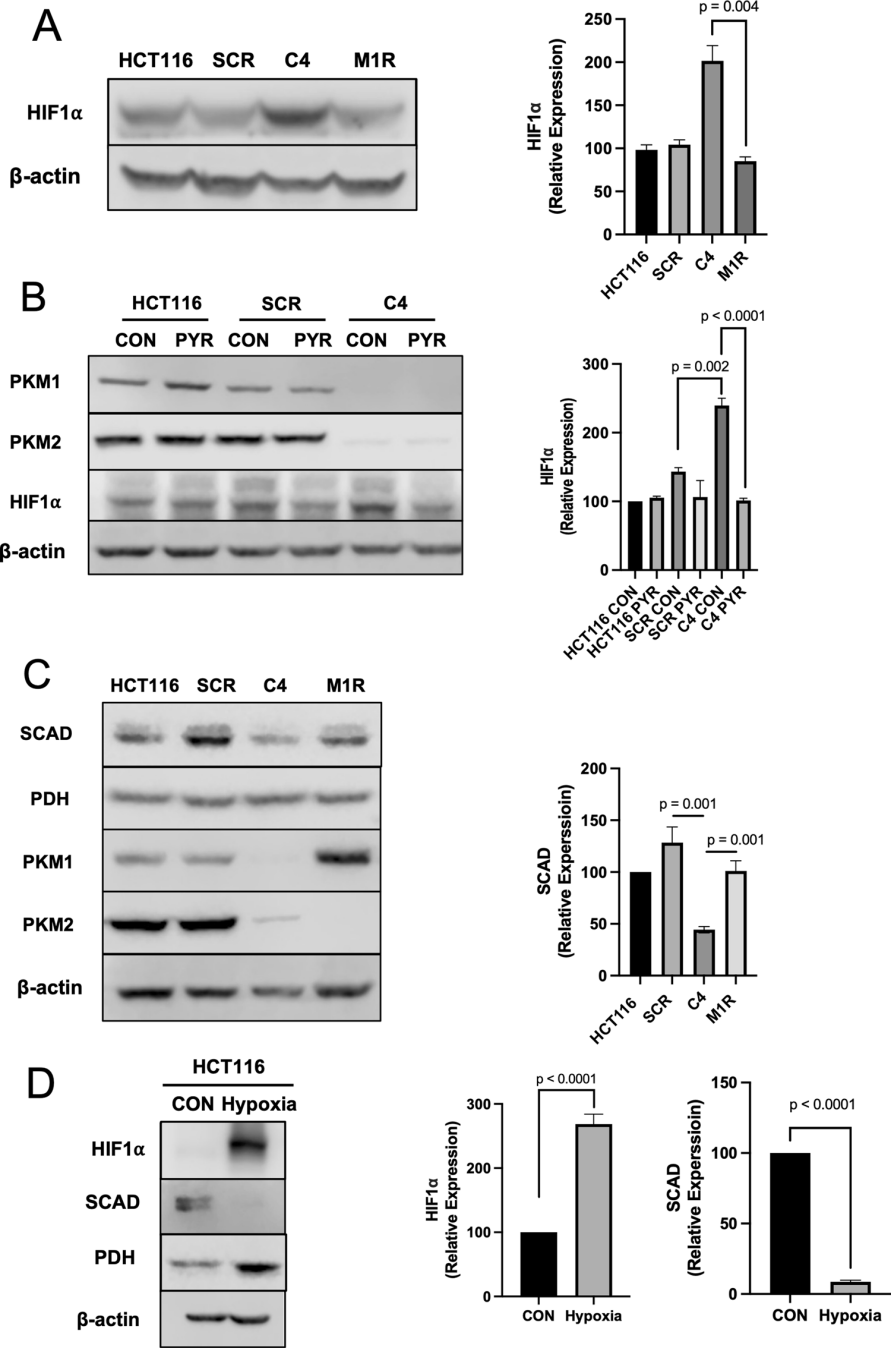
The expression of HIF1α and SCAD is affected by PKM1. (**A**) Western blot analysis of HIF1α levels in HCT116, SCR, C4, and M1R cells with β-actin as a loading control. The right panel shows the quantification of HIF1a expression. (**B**) Western blot analysis of HIF1α levels in HCT116, SCR, and C4 cells treated with and without pyruvate (5 mM). β-actin served as a loading control. (**C**) Western blot analysis of SCAD levels in HCT116, SCR, C4, and M1R cells with PDH as loading controls. (**D**) Western blot analysis of HIF1α and SCAD levels in HCT116 cells with and without hypoxic conditions for 24 h. β-actin served as loading control for HIF1α, and PDH served as loading control for SCAD. For statistical analysis, western blot was conducted 5 times. Error bars are mean ± SEM.

Hypoxia-inducible factor 1 alpha (HIF1α) is a transcription factor that promotes glycolysis in cells. Consistent with C4 cells attempting to upregulate glycolysis, HIF1α is increased. However, re-expression of PKM1 in M1R cells reduced HIF1α back to scrambled control cell levels (Fig. 6A). Based on these data and the role of pyruvate kinase in regulating the conversion of phosphoenolpyruvate (PEP) to pyruvate in the glycolytic pathway, we next sought to test whether HIF1α levels could also be reduced in C4 cells through the addition of pyruvate. Importantly, the DMEM media we use to grow the cells and in some of the experiments lacks pyruvate. The addition of pyruvate to the media decreased HIF1α in C4 cells as compared to scramble control cells (Fig. 6B). This alludes to the lack of pyruvate through the diminishment of PKM1 in C4 cells as the primary factor in the upregulation of HIF1α. Concomitant with the increase in HIF1α observed in C4 cells, short-chain acyl dehydrogenase (SCAD) was found to be decreased. SCAD mediates butyrate oxidation, and therefore any diminishment in this enzyme would result in a decrease in butyrate oxidation. Re-expression of PKM1 in M1R cells increased SCAD levels (Fig. 6C). SCAD is the first enzyme required for short-chain fatty acids (SCFAs), including butyrate, oxidation in the mitochondria^32^. Furthermore, when HCT116 cells were cultured under a hypoxic condition for 24 h to induce HIF1α, we found that SCAD levels were significantly diminished (Fig. 6D). These data suggested that loss of PKM1 in C4 cells resulted in the cells attempting to increase glycolysis, likely through diminished pyruvate levels, and a reduction in SCAD levels was observed in conjunction with the increase in HIF1α. An increase in HIF1α and a decrease in SCAD may underlie lower butyrate oxidation observed in C4 cells.

### Knockout of HIF1α increases butyrate oxidation in colorectal cancer cells

**Figure 7:**
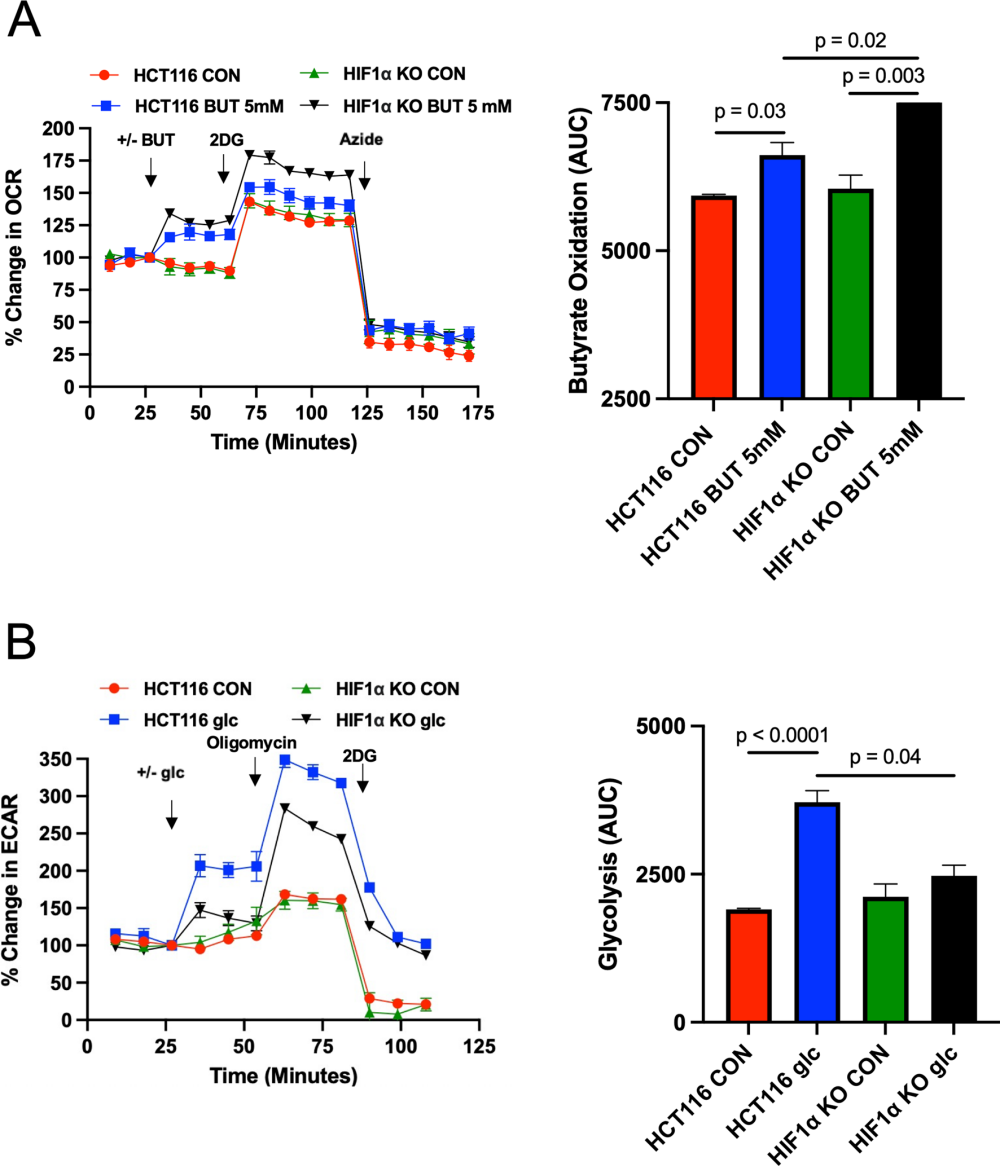
The role of HIF1α in regulating butyrate oxidation. (**A**) Percent change in oxygen consumption rate (OCR) relative to baseline in which HCT116 and HIF1α KO cells treated with and without butyrate (5 mM). Total contribution of butyrate toward OCR (%) is observed after injection of 2-deoxyglucose (2DG). The right panel shows the area under the curve (AUC) analysis from OCR measurements taken after 2DG injection but before azide injection. These measurements represent butyrate oxidation (arbitrary units). Data points represent the average OCR (%) over 3–5 replicates per condition for butyrate oxidation measurements. (**B**) Percent change in extracellular acidification rate (ECAR) relative to baseline in which HCT116 and HIF1α KO cells respond to glucose, 2DG, and azide. The right panel shows AUC from ECAR measurements taken after glucose injection but before 2DG injection. These measurements represent glycolysis (arbitrary units). Data points represent the average ECAR (%) over 3–5 replicates per condition for glycolysis measurements. Error bars are mean ± SEM.

To test whether HIF1α plays a role in regulating butyrate oxidation, we obtained a HIF1α knockout cell line (HIF1α −/−)^33^, which was derived from a parental HCT116 cell line. HIF1α was indeed absent in this cell line (Supplemental Fig. 6). In line with HIF1α, suppressing oxidative metabolism, we found that knockout of HIF1α resulted in an increase in the oxidation of butyrate (Fig. 7A). Moreover, CRC cells that lack HIF1α showed a diminishment in glycolysis as judged by the decreased extracellular acidification rate (ECAR) after the addition of glucose (Fig. 7B). ECAR is used as an indirect measurement of lactate production, which is an indication of glycolysis. Taken together, these data allude to lower PKM1 levels in stabilizing HIF1α, which subsequently results in increased glycolysis and decreased butyrate oxidation in CRC cells.

## Discussion

The data surrounding PKM2 and colorectal cancer (CRC) is controversial. Originally, studies seemed to show a role for PKM2 in promoting glycolysis and CRC^14,15,34^. However, more recent studies have found either no role for PKM2 or the opposite result toward suppressing CRC^35,36^. In one study, loss of PKM2 in colonic stem cells increased colorectal tumorigenesis in an inflammation-associated colorectal tumor mouse model^36^. This would suggest that PKM2 has tumor suppressor activity as opposed to being a proto-oncogene. Compared to PKM2, little to nothing is known in regard to PKM1 as it relates to colorectal cancer. In a carcinogen-induced small lung cancer mouse model, PKM1 was reported to be the main important factor in tumor growth, development, and malignancy^37^. The data presented here also support a role for PKM1, as opposed to PKM2, in colorectal cancer. However, in contrast to PKM1 promoting small lung cancer, our data suggest that PKM1 may play an inhibitory role in colorectal cancer as human carcinoma biopsies showed very little PKM1 compared to non-cancerous colorectal tissue.

Colorectal cancer cells that are significantly diminished in PKM1 and PKM2 continue to grow and proliferate, albeit slower than colorectal cancer cells that express both of these isoforms at full levels. As such, we found that these PKM1 and PKM2 deficient cells show a major shift in metabolism. Going into these experiments, it was expected that PKM2 was the major isoform promoting glycolysis, whereas PKM1 would promote oxidative metabolism. Thus, in initial experiments, where two CRC clone cell lines both lacked PKM2 (C4 and C5), the opposing results in terms of butyrate oxidation were surprising and provided the first hint that other factors besides PKM2 may be important for regulating cell metabolism in regard to butyrate oxidation. Importantly, PKM1 expression (C4 PKM1 was minimally detected by western blot, and C5 PKM1 was overexpressed) was significantly different among the clones. Thus, it became important to test whether butyrate oxidation could be rescued through re-expressing the PKM1 isoform in the C4 cells. In fact, the addition of PKM1 increased butyrate oxidation and provided evidence that it is an important player in the oxidation of butyrate in colorectal cancer cells. However, we cannot exclude the involvement of PKM2 in regulating butyrate oxidation, although PKM1 appears to have a much greater regulatory role in positively impacting this process. Moreover, it has been suggested that butyrate oxidation is decreased in colorectal cancer cells^38^, which is in line with the diminished PKM1 expression in colorectal cancer biopsies. These results also support other studies defining a function of PKM1 toward regulating and promoting oxidative metabolism in cells^39^.

Regarding this role in regulating oxidative metabolism, PKM1, but not PKM2, has been reported to localize to the mitochondria in H1299 and A549 lung cancer cell lines^31^. In addition, stable knockdown of the PKM1/2 isoforms activated AMPK in these cells, suggesting that loss of these isoforms results in energetic stress^31^. In the colorectal cancer cells used in our study, it was determined that loss of both isoforms resulted in AMPK activation, which was rescued by the re-addition of PKM1. Since colorectal cancer cells deficient in PKM1 and PKM2 showed reduced butyrate oxidation that was PKM1 dependent, it was assumed that these cells would compensate, perhaps through increasing glycolysis.

**Figure 8:**
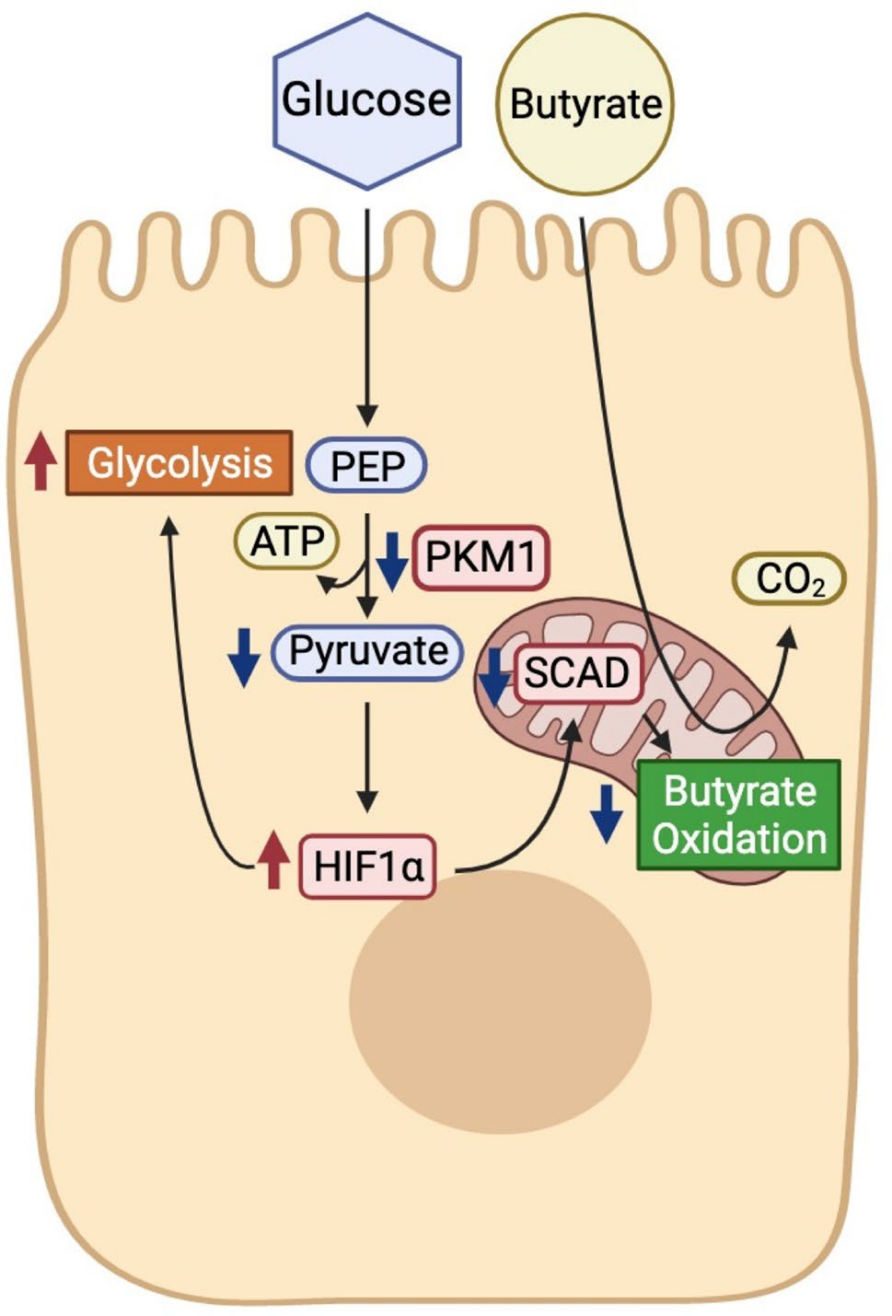
Model of PKM1 regulation of cellular metabolism in cancerous colonocyte. Working model of pathways regulating butyrate oxidation. In this model, the downregulated metabolic enzyme PKM1 is a key factor in the shift of cancerous colonocytes from oxidative metabolism toward aerobic glycolysis. The loss of PKM1 in cancerous colonocytes leads cells to increase glycolysis through reduction of SCAD along with an increase in HIF1α due to a decrease in pyruvate levels. The increase in HIF1α and the decrease in SCAD together explain the lower butyrate oxidation in CRC cells.

Taken together, our data indicate that the diminishment of PKM1, rather than increased PKM2, is a key factor in the metabolic shift of CRC cells by reducing oxidative metabolism and promoting glycolysis. In our model, a diminishment in PKM1 is a mechanism to explain the metabolic shift in CRC cells (Fig. 8). A decrease in PKM1 causes an increase in HIF1α through reduced concentration of pyruvate, resulting in lower SCAD expression, and ultimately suppressing butyrate oxidation and promoting glycolysis.

## Methods

### Cell culture and shRNA transfection

The human colon cancer cell line HCT116 (ATCC, CCL-247) was grown in DMEM supplemented with 25 mM glucose and 10% FBS. PKM1 and PKM2 silencing in HCT116 was achieved using five different hairpins (GeneCopoeia, Inc.; Rockville, MD). Lentivirus packaging system (GeneCopoeia) was used to generate the shRNA-containing lentivirus particles in HEK293FT cells (GeneCopoeia) following the manufacturer’s guidelines and then used to infect HCT116. Two scrambled non-silencing shRNA were used as controls. Cells were selected using puromycin (2 μg/ml). For PKM1 and PKM2 overexpression, colorectal cells were infected with EX-Lv244-mCherry-PKM1 or EX-Lv122-eGFP-PKM2 lentiviruses (GeneCopoeia) respectively, selected using a combination of puromycin (2 μg/ml) and hygromycin (200 μg/ml), and propagated.

### Flux experiments

To measure butyrate oxidation and mitochondrial function, XFe24 Extracellular Flux Analyzer (Seahorse Bioscience) was used. All Seahorse assay experiments were conducted following the manufacturer’s guidelines. The % change of the oxygen consumption rates (OCR) after butyrate injection was measured. Cells were seeded at 4.5 × 10^5^/well in XFe24 cell culture microplates (Agilent Technologies, 100850). One hour before measurement, the culture medium was replaced with 1× Krebs–Henseleit Buffer (KHB) (5 mM glucose and 500 μM carnitine) for the butyrate oxidation analysis and with Seahorse XF DMEM medium (5 mM glucose, 2 mM glutamine, and 1 mM pyruvate) for the mitochondrial function test, and the cell plate was incubated in a non-CO_2_ incubator at 37 °C for 1 h. All Seahorse experiments were run with identical conditions (unless otherwise noted). Briefly, for the butyrate oxidation analysis, 1× KHB media or sodium butyrate (Sigma, B5887) at 5 mM final concentration were injected, and the change in OCR was measured from baseline (%OCR). Next, 2-deoxyglucose (2DG) 50 mM (Sigma, D8375) was injected to competitively inhibit glucose utilization and leave butyrate as the only exogenous energy substrate. Finally, 10% sodium azide was injected to block mitochondrial respiration by inhibiting complex IV. For the mitochondrial function test, 1× KHB media or sodium butyrate (Sigma, B5887) at 5 mM final concentration were treated 15 min before measurement. Then, Oligomycin 1 μM (Alfa Aesar, AAJ61898MA) was injected into the wells to inhibit ATP synthase (complex V) following basal measurement. Next, Carbonyl cyanide-4 (trifluoromethoxy) phenylhydrazone (FCCP) 10 μM (Cayman Chemical, NC0904863), which is an uncoupling agent that collapses the proton gradient and disrupts the mitochondrial membrane potential, was injected into the wells. At last, a mixture of rotenone 1 μM (Enzo Life Science, 03-1755), a complex I inhibitor, and antimycin A 1 μM (Sigma Aldrich, A8674), a complex III inhibitor were injected into the wells to shut down mitochondrial respiration and calculate the nonmitochondrial respiration. After the measurements, cells were lysed with 1× RIPA buffer (Cell Signaling, 9806s), and proteins were quantified using Pierce BCA Protein Assay Kit (Thermo Fisher, PI23228) for normalization.

### Western blot analysis

Proteins from cell lines were extracted with 1× RIPA buffer (Cell Signaling, 9806s), 1 mM PMSF (Cell Signaling, 8553), 0.5 M EDTA Solution (Thermo Scientific, R1021), and Halt™ Phosphatase Inhibitor Cocktail (Thermo Scientific, PI87786). Samples were spun in a centrifuge at 13,000×*g* for 10 min at 4 °C, and protein supernatant was transferred to a new 1.5 ml Eppendorf tube. Protein concentrations were determined by Pierce BCA Protein Assay Kit (Thermo Fisher, PI23228). Proteins were separated on 8 and 10% SDS–polyacrylamide gels and transferred onto the PVDF membrane. Membranes were incubated on a rotator in 5% BSA in 1× TBST (0.1%) for 1 h at room temperature (RT). Blocked membranes were incubated overnight at 4 °C with the respective primary antibodies on a rotator. Antibodies that were used included PKM1 (Cell Signaling, 7067), PKM2 (Cell Signaling, 4053s), HIF1α (Cell Signaling, 3716s), SCAD (Abcam, ab154823), AMPKα (Cell Signaling, 5831), phospho-AMPKα (Thr172) (Cell Signaling, 2535), PDH (Cell Signaling, 3205), phospho-PDHA1 (Abcam, ab92696), and ß-actin (Cell Signaling, 3700). Blots were washed 3 times for 10 min/wash in 1× TBST (0.1%) at RT and incubated with fluorescent secondary antibody for 2 h on rotator at RT following 3 times for 10 min/wash in 1× TBST (0.1%) at RT. Fluorescent detection or chemiluminescence detection of PVDF membrane was performed with the Odyssey Fc and bands were quantified with Image Studio Software (LI-COR Biosciences, Lincoln, NE).

### Immunofluorescence staining

The clinical samples were obtained from the University of Tennessee Medical Center Biobank with IRB# 4044. Human normal and tumor colon tissues were fixed with neutral buffered 10% formalin for 24–36 h, dehydrated with 70% to 100% ethanol and processed for the production of 4-μm paraffine sections. Formalin-fixed paraffin-embedded tissue sections were deparaffinized and rehydrated with xylene, ethanol, and PBS. The rehydrated sections were blocked with 1% BSA and 5% Horse serum in PBS for 1 h at room temperature (RT), and stained with PKM1 (Cell Signaling, 7067) and PKM2 (Cell Signaling, 4053s) primary antibodies overnight at 4 °C. Tissue sections were washed in PBS, incubated with donkey anti-mouse IgG (H + L) Texas Red secondary antibodies at RT for 1 h, and mounted with Prolong Gold Antifade Mountant with DAPI (Thermo Scientific, P36931) and coverslips. Tissues were analyzed with LEICA DMi8 microscope equipped with FITC, DAPI, Texas Red, and EMP_BF filters. All images were acquired with the same exposure and focal plane. Integrated density (total fluorescent) per cell was quantified by the Image J (Bethesda, MD) program.

### Statistical analysis

For seahorse experiments, western blotting, and immunofluorescence, ANOVA was used to test for differences between experimental groups followed by a Tukey post-hoc test. All data are expressed as mean ± SEM.

## Supplementary Information

Supplementary Figure 1.

Supplementary Figure 2.

Supplementary Figure 3.

Supplementary Figure 4.

Supplementary Figure 5.

Supplementary Figure 6.

Supplementary Figure 7.

Supplementary Figure 8.

## Data Availability

All generated raw data and/or analyzed data from the current study are available from the corresponding author on reasonable request.

## Abbreviations

PKM1: Pyruvate kinase M1
PKM2: Pyruvate kinase M2
PEP: Phosphoenolpyruvate
SCFA: Short-chain fatty acid
PDH: Pyruvate dehydrogenase complex
HIF1α: Hypoxia-inducible factor 1 alpha
HDAC: Histone deacetylase
SCAD: Short-chain acyl dehydrogenase
KHB: Krebs–Henseleit buffer
OCR: Oxygen consumption rate
ECAR: Extracellular acidification rate
2DG: 2-Deoxyglucose
