# Supplementary figures and images for: Pyruvate kinase M1 regulates butyrate metabolism in cancerous colonocytes
Source: Sci Rep. 2022 May 24;12:8771. doi: 10.1038/s41598-022-12827-9 (PMC9130307; doi:10.1038/s41598-022-12827-9)

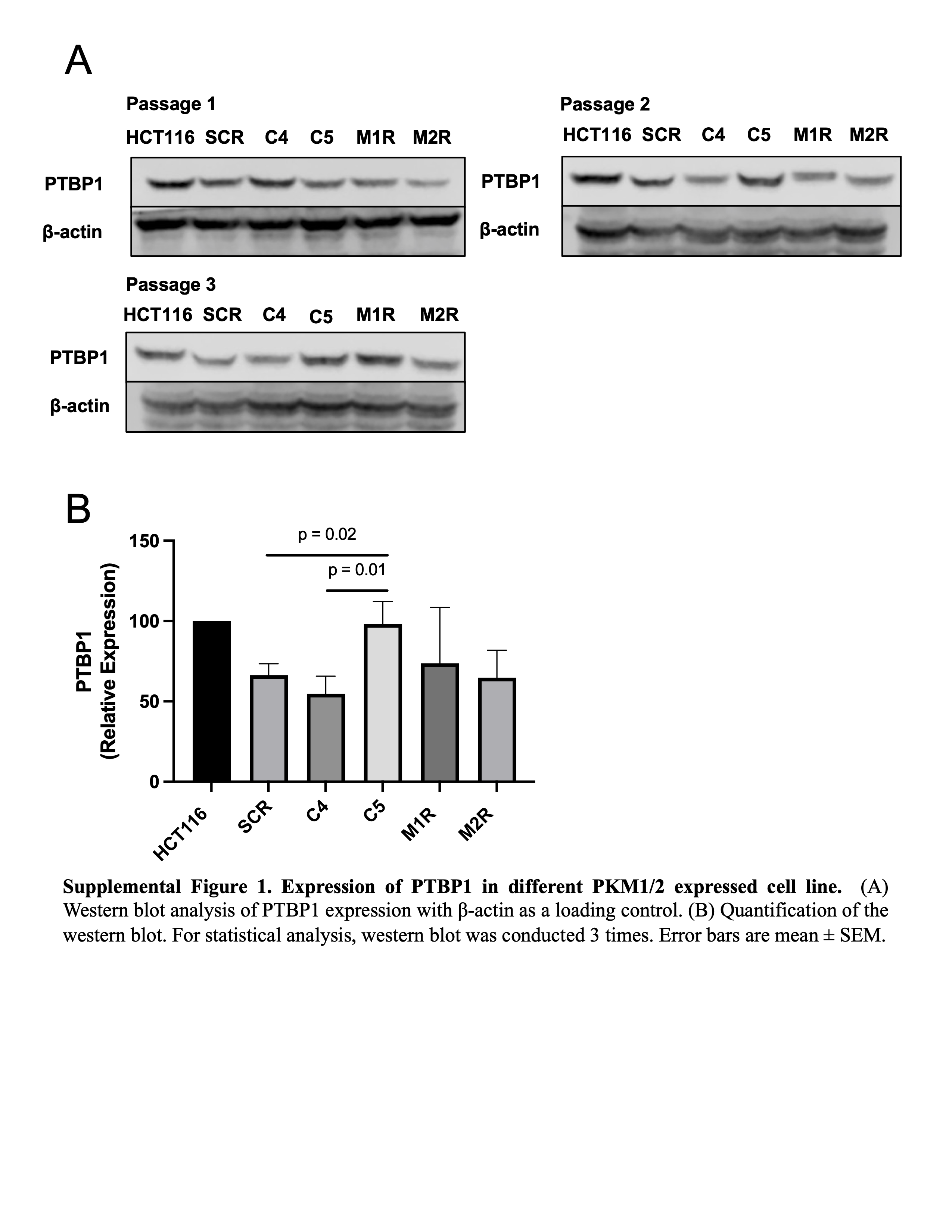

Supplement: Supplementary file 1 — Supplementary Figure 1. [file 41598_2022_12827_MOESM1_ESM.tiff]

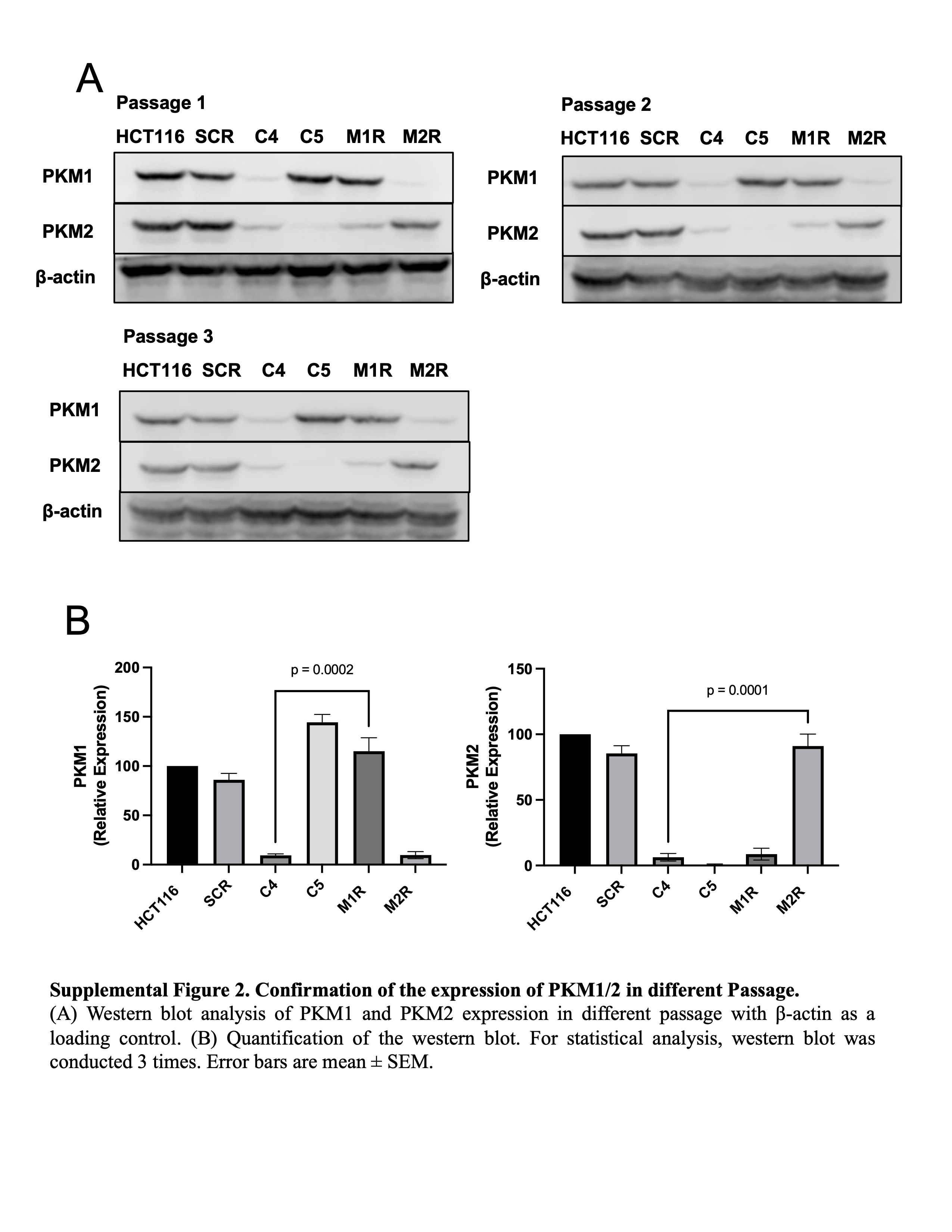

Supplement: Supplementary file 2 — Supplementary Figure 2. [file 41598_2022_12827_MOESM2_ESM.tiff]

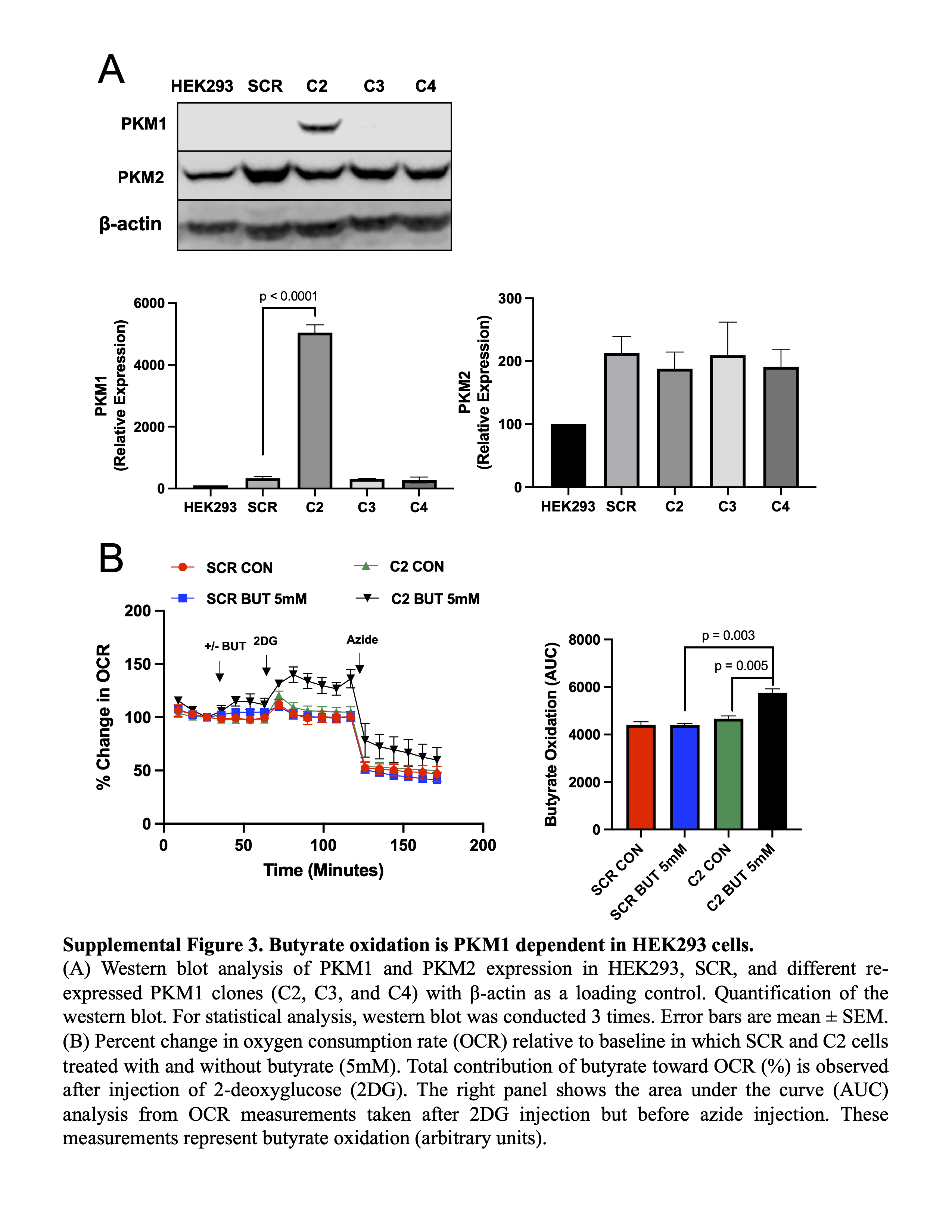

Supplement: Supplementary file 3 — Supplementary Figure 3. [file 41598_2022_12827_MOESM3_ESM.tiff]

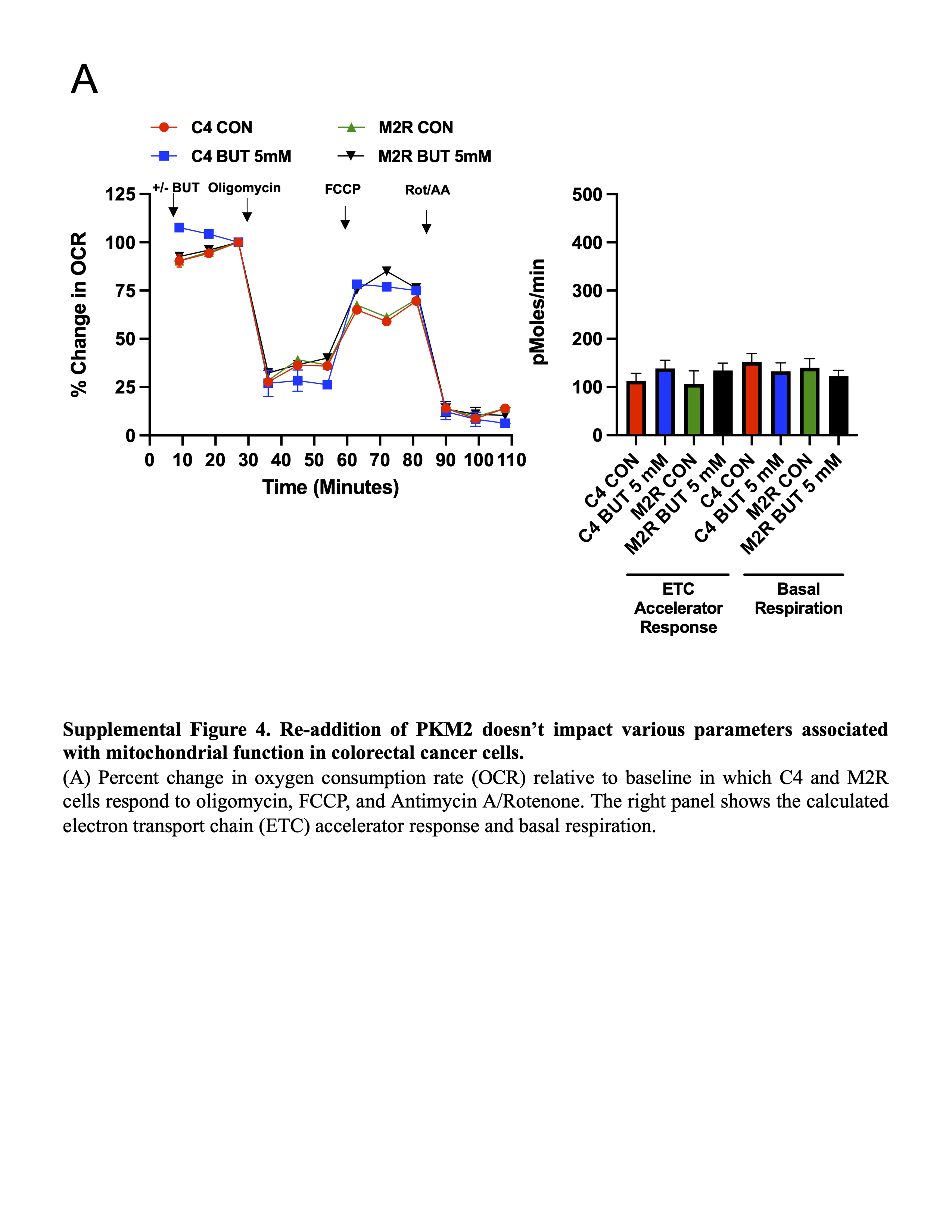

Supplement: Supplementary file 4 — Supplementary Figure 4. [file 41598_2022_12827_MOESM4_ESM.tiff]

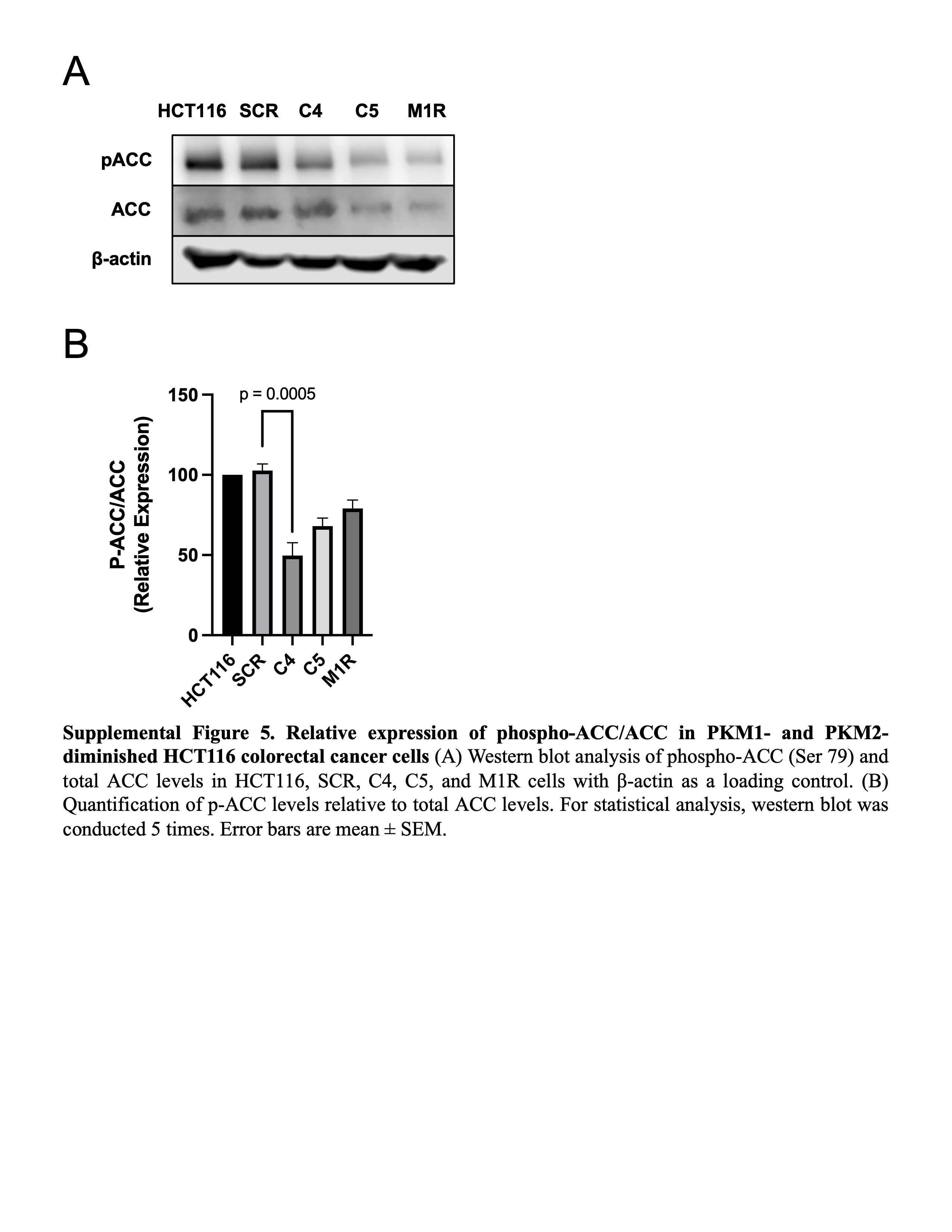

Supplement: Supplementary file 5 — Supplementary Figure 5. [file 41598_2022_12827_MOESM5_ESM.tiff]

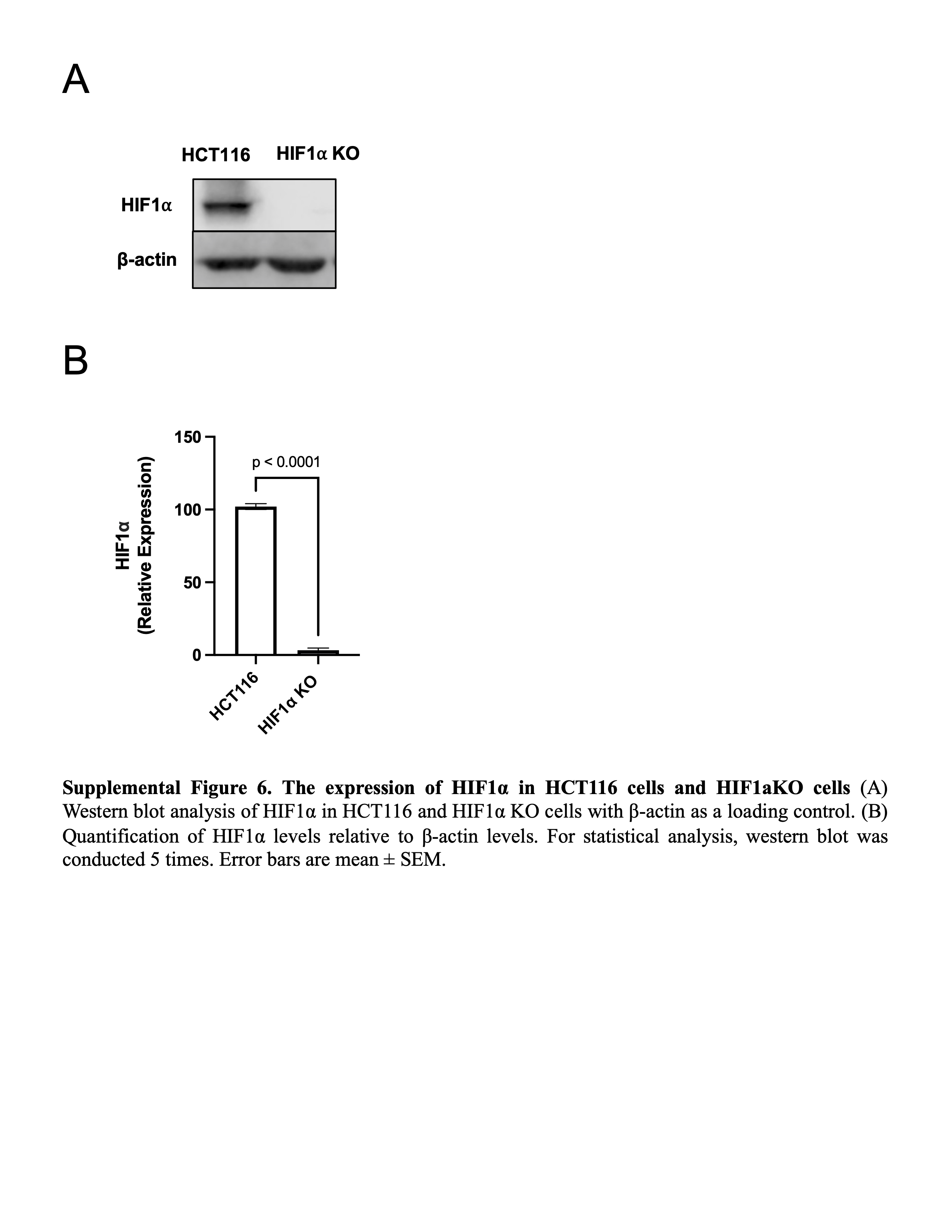

Supplement: Supplementary file 6 — Supplementary Figure 6. [file 41598_2022_12827_MOESM6_ESM.tiff]
