# Supplementary material for: Pyruvate kinase M1 regulates butyrate metabolism in cancerous colonocytes
Source: Sci Rep. 2022 May 24;12:8771. doi: 10.1038/s41598-022-12827-9 (PMC9130307; doi:10.1038/s41598-022-12827-9)
Supplement: Supplementary file 7 — Supplementary Figure 7. [file 41598_2022_12827_MOESM7_ESM.pdf]

Figure 2A

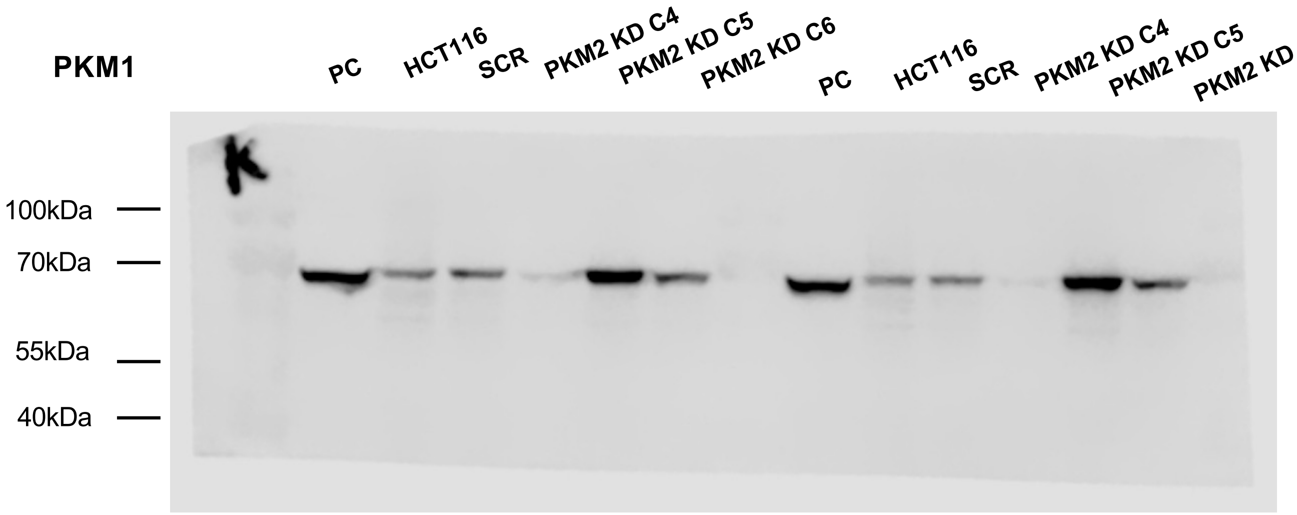

*Membrane was cut before primary Ab incubation.

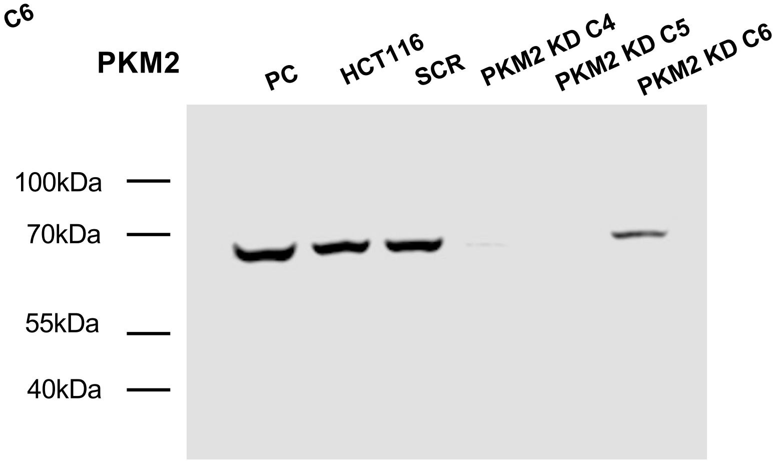

*Membrane was cut before primary Ab incubation.

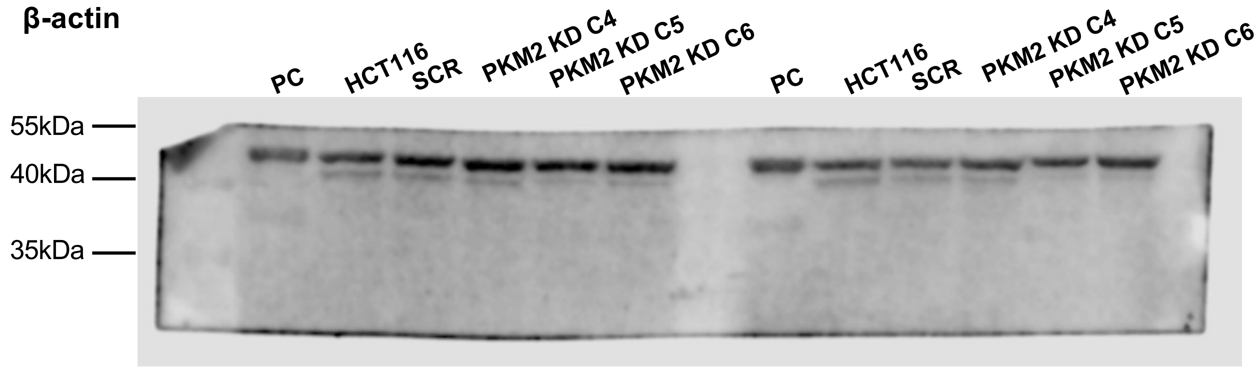

*Membrane was cut before primary Ab incubation.

Figure 3A

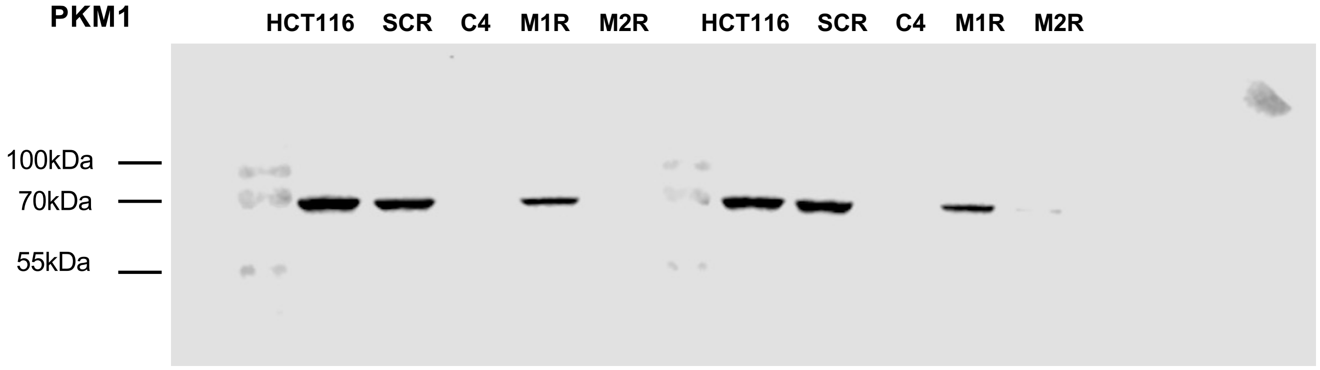

*Membrane was cut before primary Ab incubation.

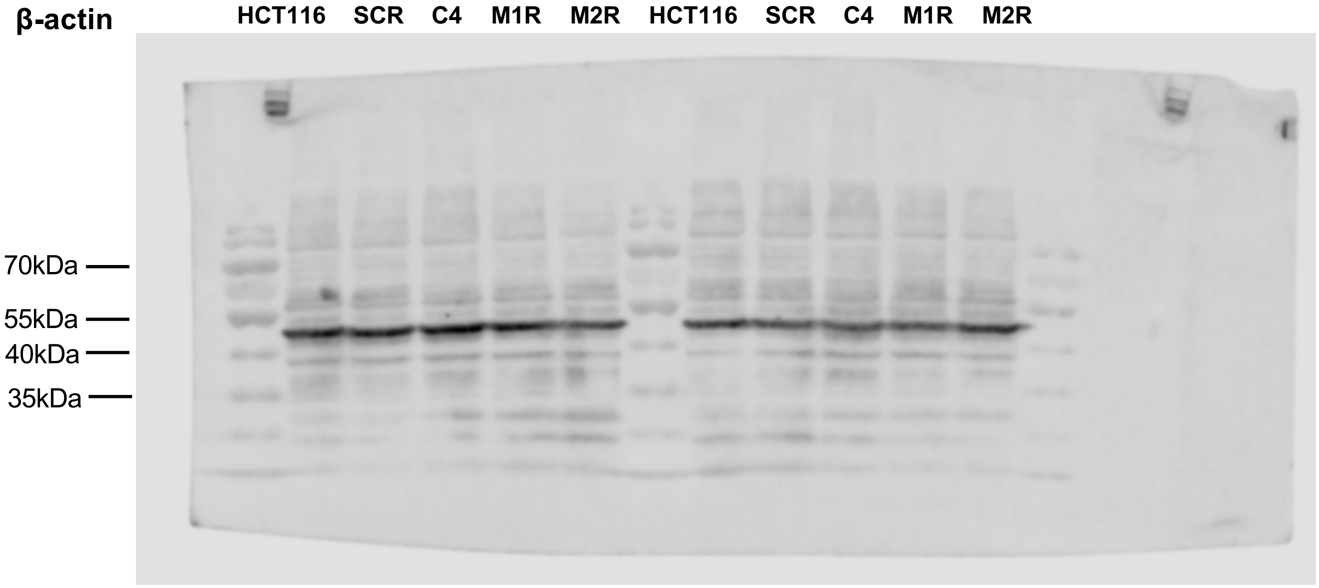

*Membrane was cut before primary Ab incubation.

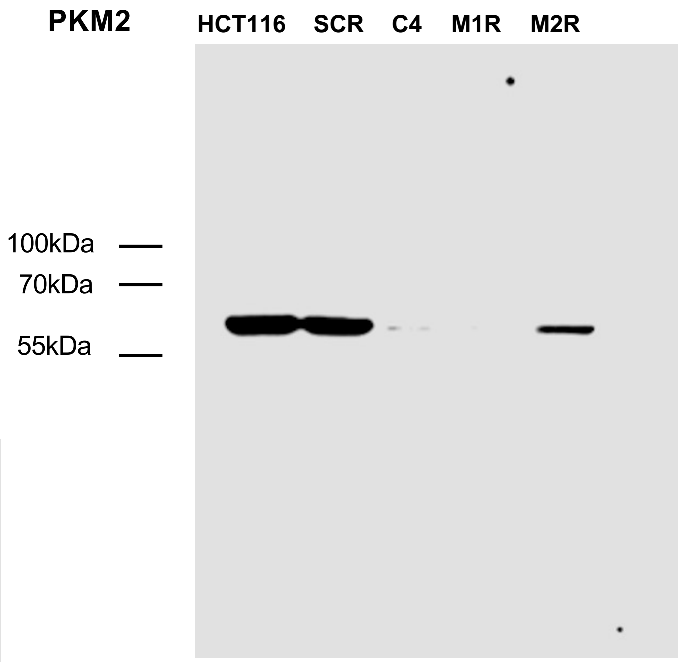

*Membrane was cut before primary Ab incubation.

Figure 5A

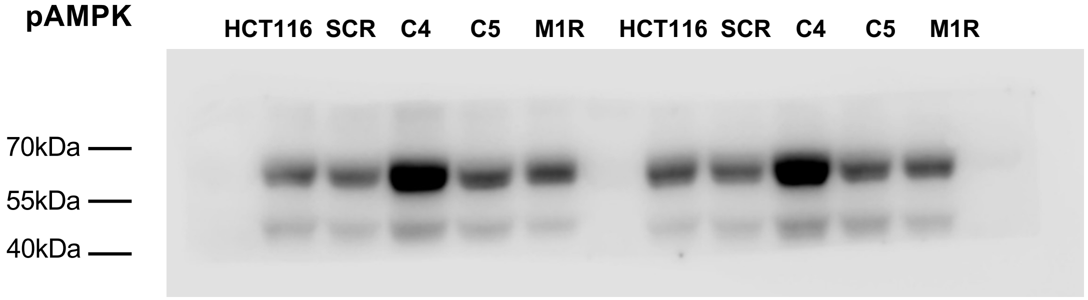

*Membrane was cut before primary Ab incubation.

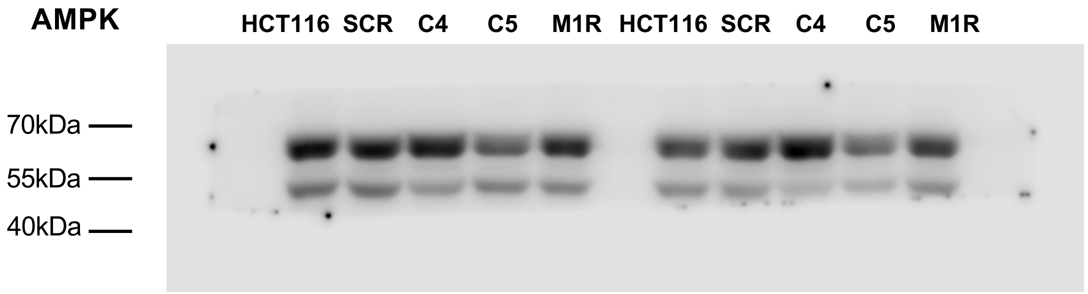

*Membrane was cut before primary Ab incubation.

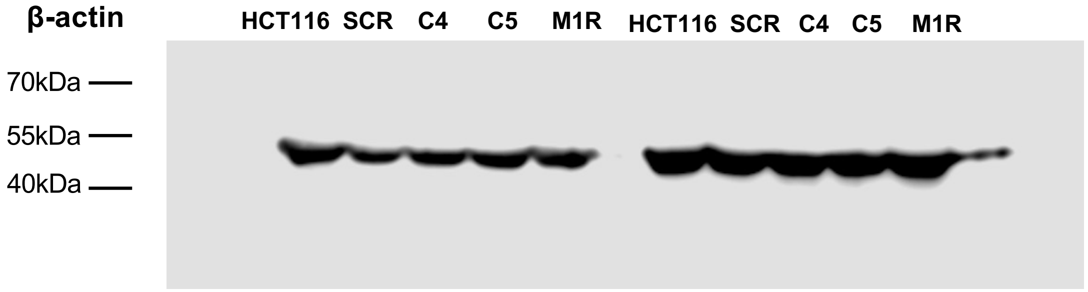

*Membrane was cut before primary Ab incubation.

Figure 5B

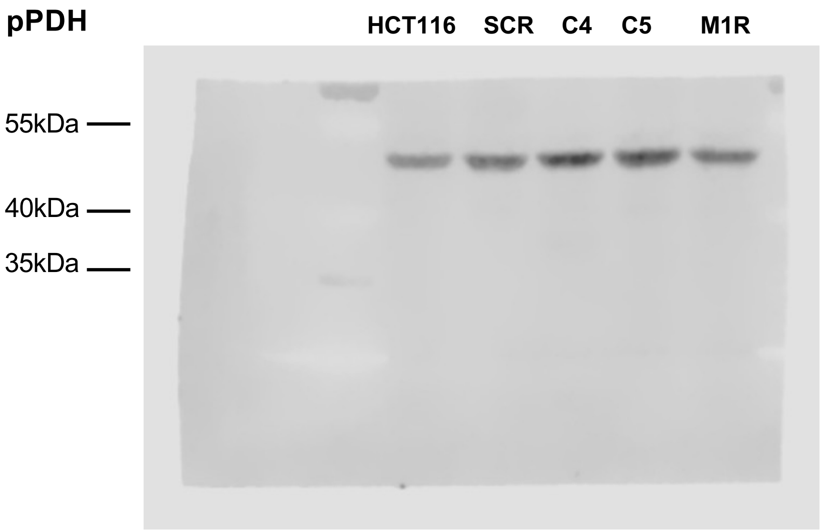

*Membrane was cut before primary Ab incubation.

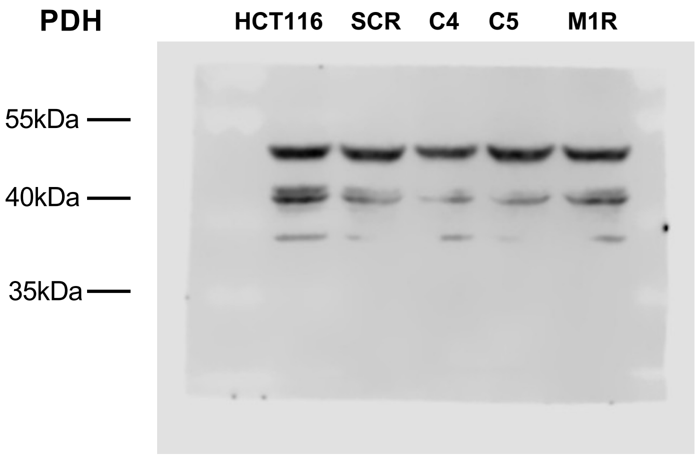

*Membrane was cut before primary Ab incubation.

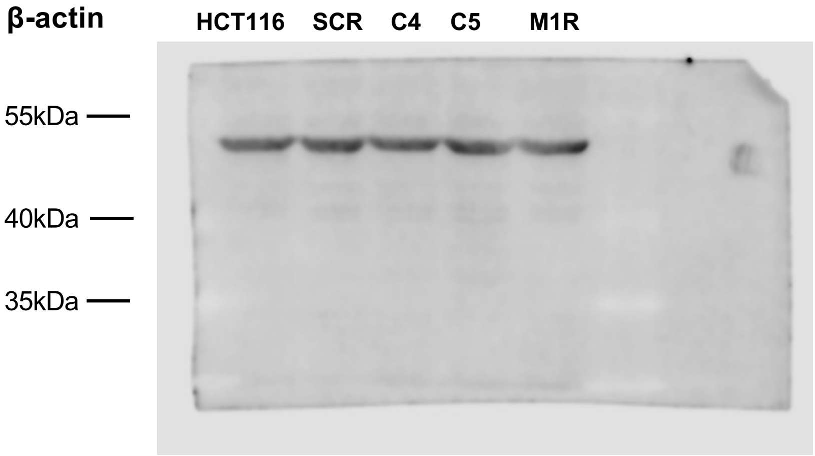

*Membrane was cut before primary Ab incubation.

Figure 6A

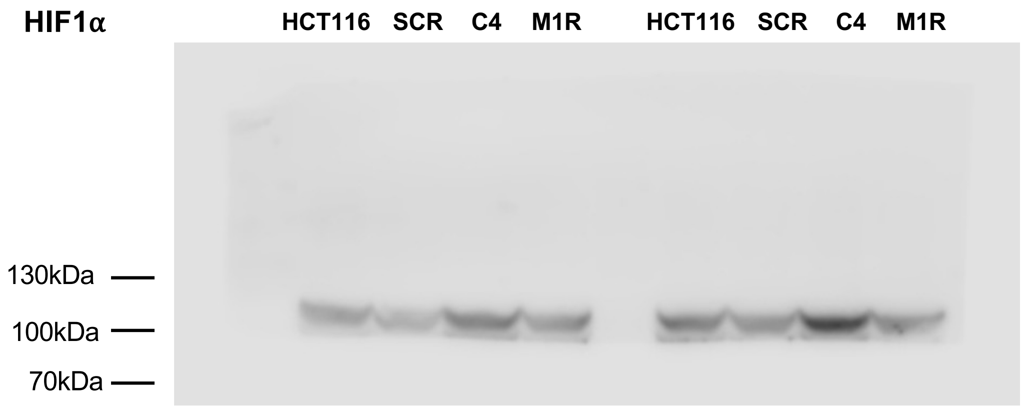

*Membrane was cut before primary Ab incubation.

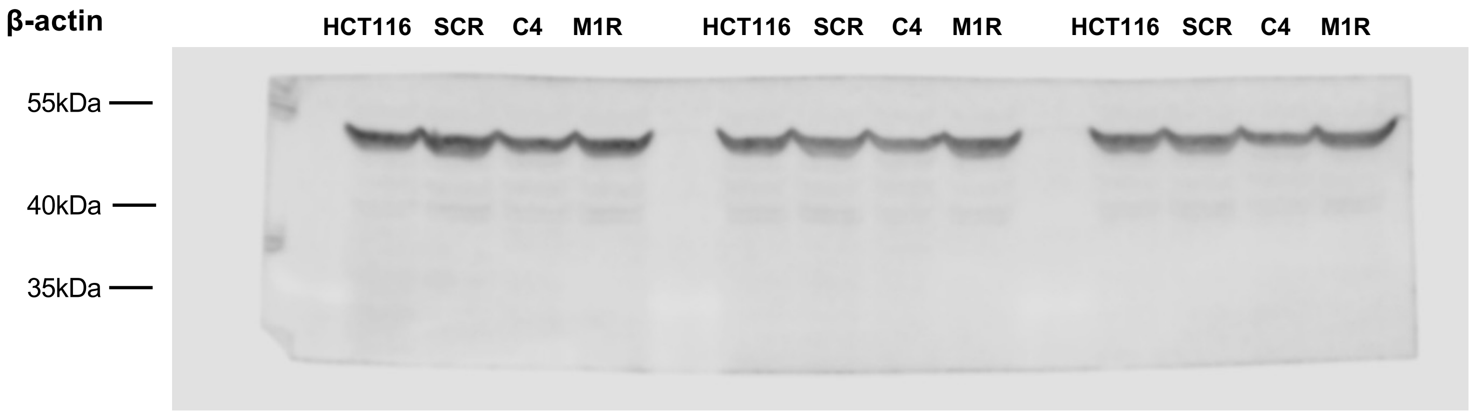

*Membrane was cut before primary Ab incubation.

Figure 6B

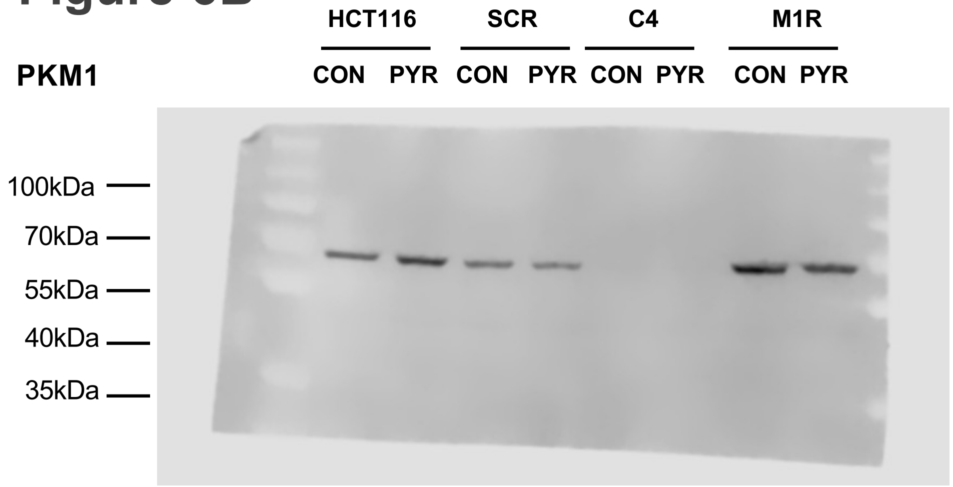

*Membrane was cut before primary Ab incubation.

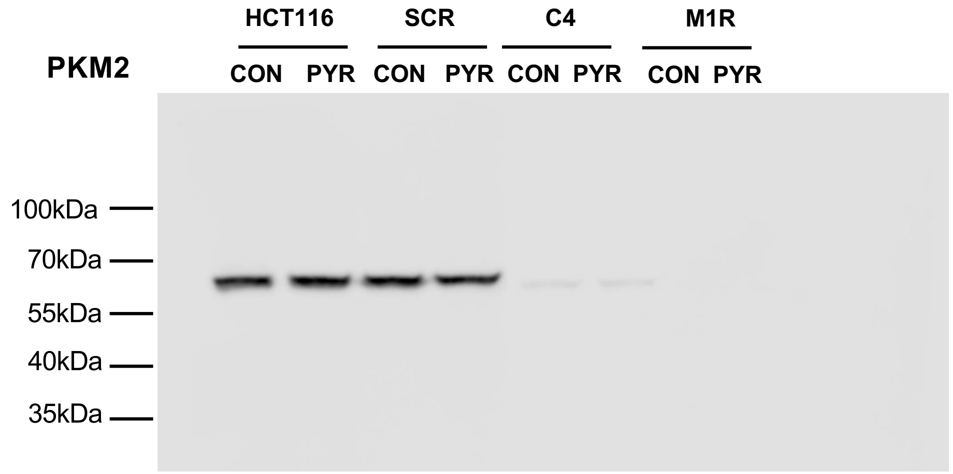

*Membrane was cut before primary Ab incubation.

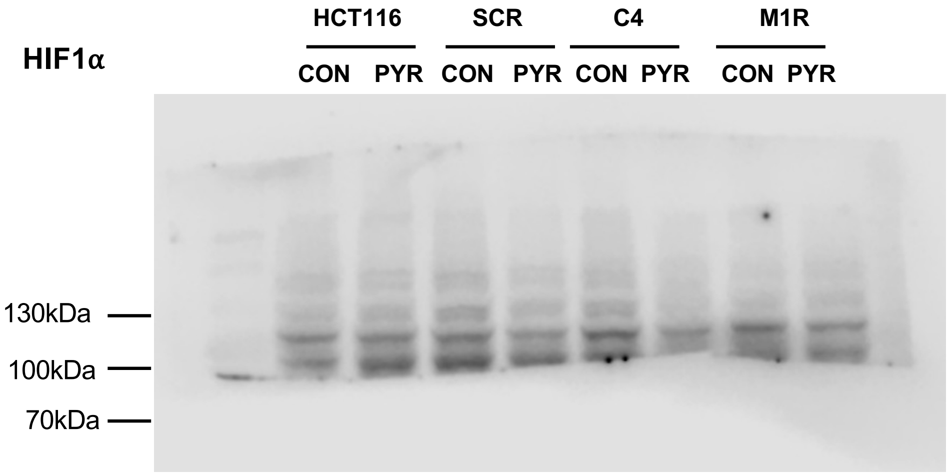

*Membrane was cut before primary Ab incubation.

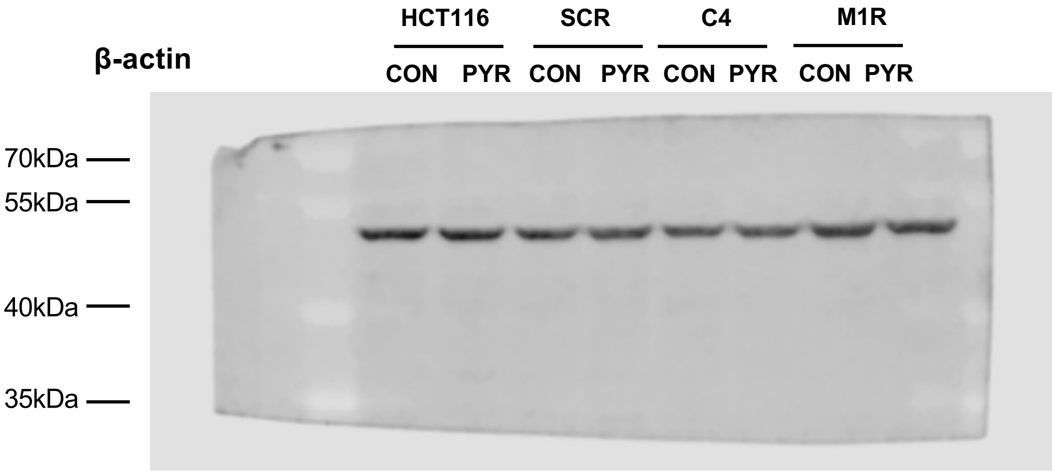

*Membrane was cut before primary Ab incubation.

Figure 6C

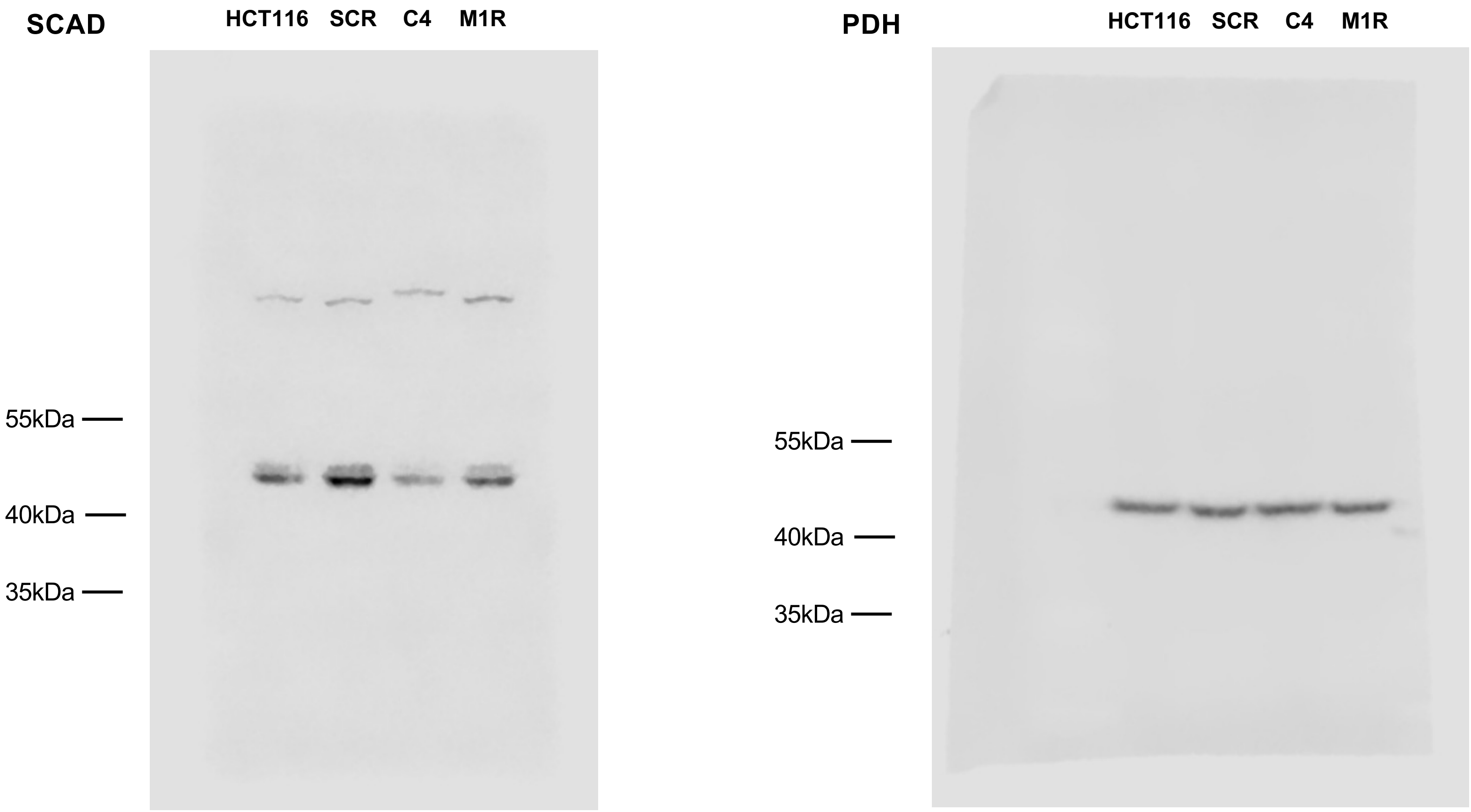

*Membrane was cut before primary Ab incubation.

*Membrane was cut before primary Ab incubation.

Figure 6C

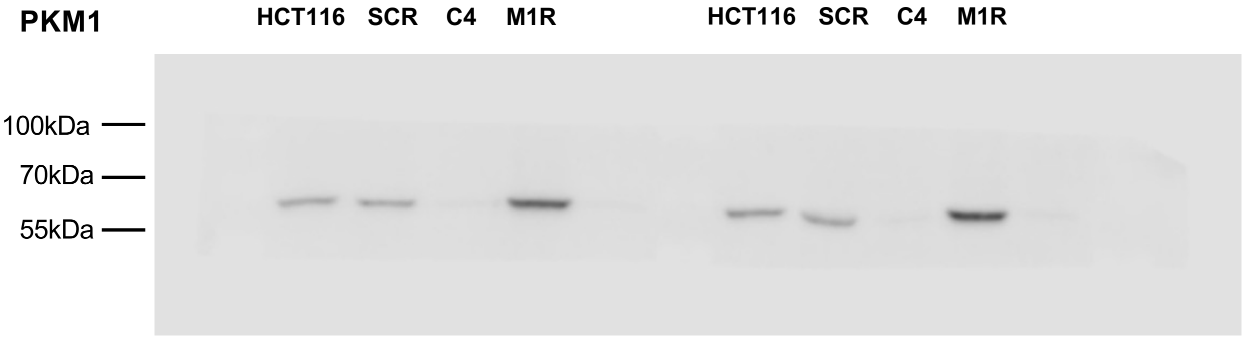

*Membrane was cut before primary Ab incubation.

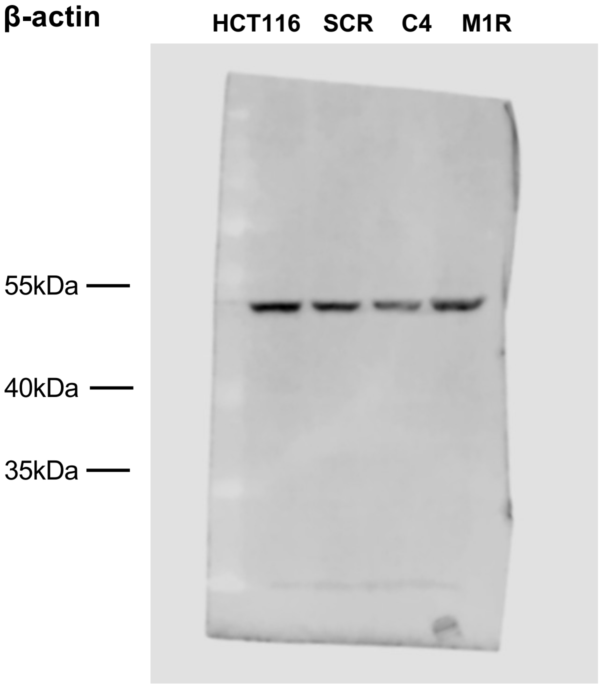

*Membrane was cut before primary Ab incubation.

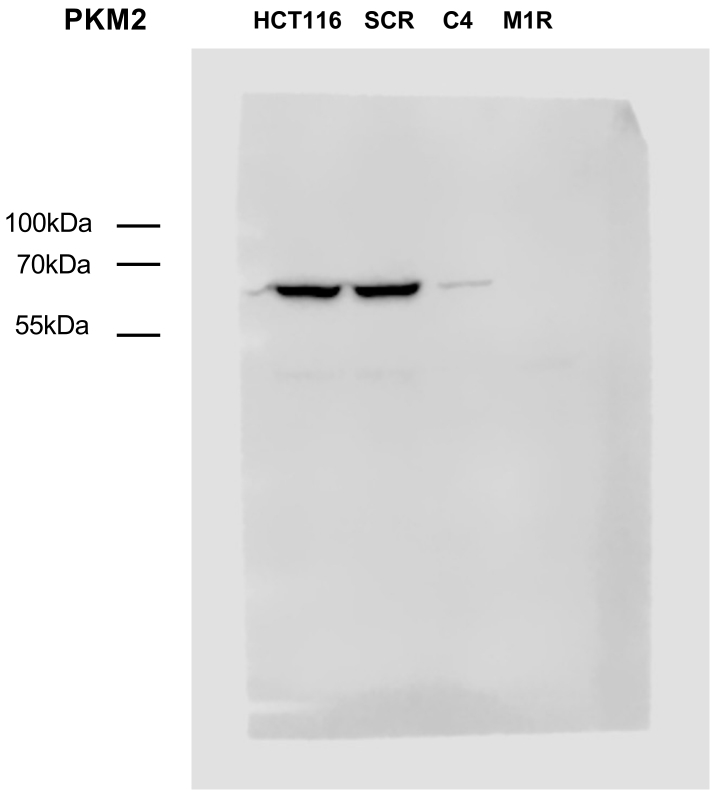

*Membrane was cut before primary Ab incubation.

Figure 6D

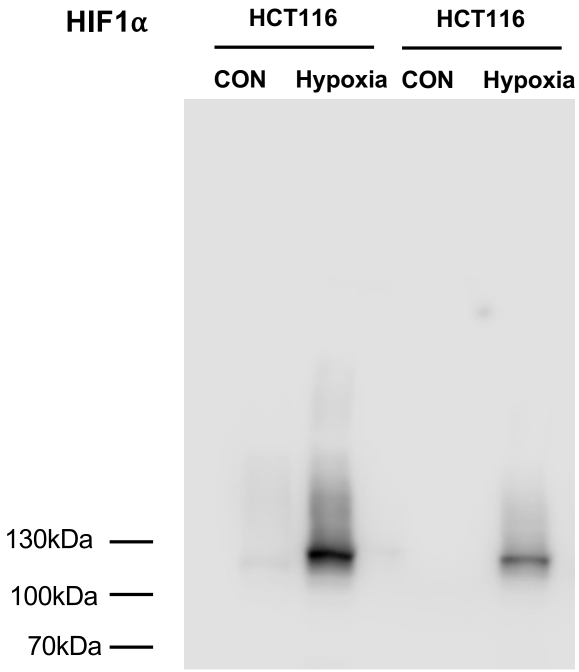

*Membrane was cut before primary Ab incubation.

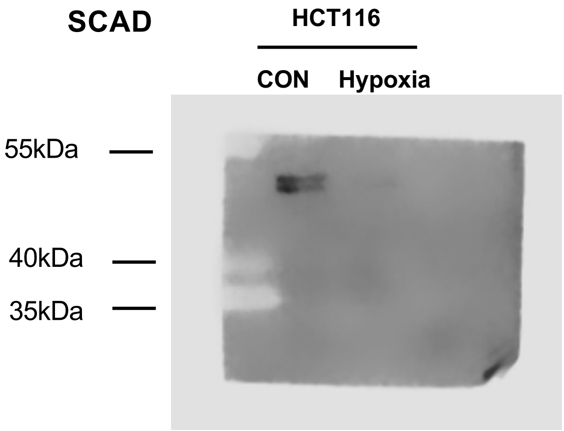

*Membrane was cut before primary Ab incubation.

Figure 6D

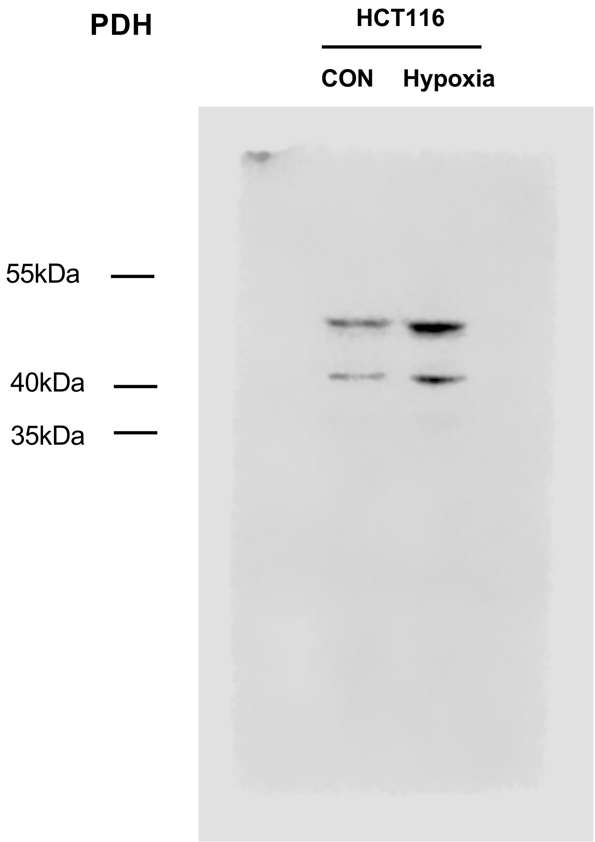

*Membrane was cut before primary Ab incubation.

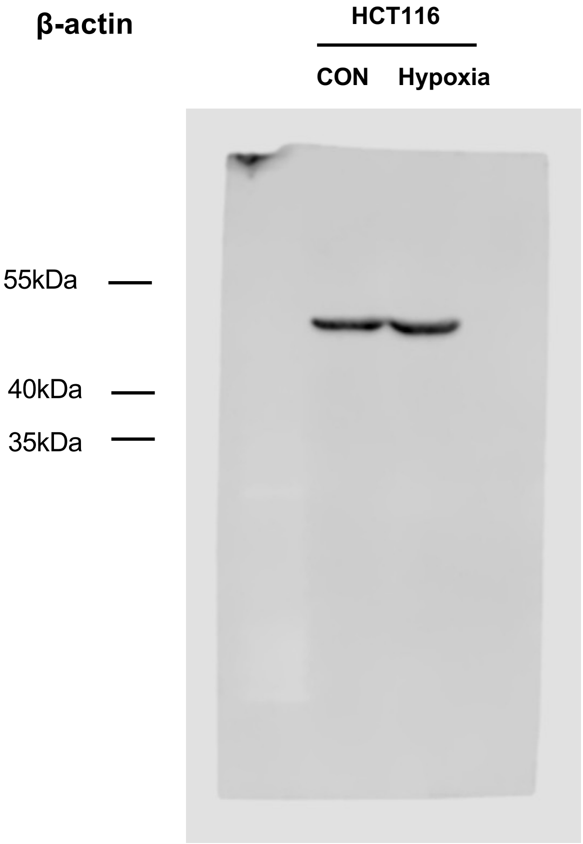

*Membrane was cut before primary Ab incubation.

Supplemental Figure 1A

Passage 1

PTBP1

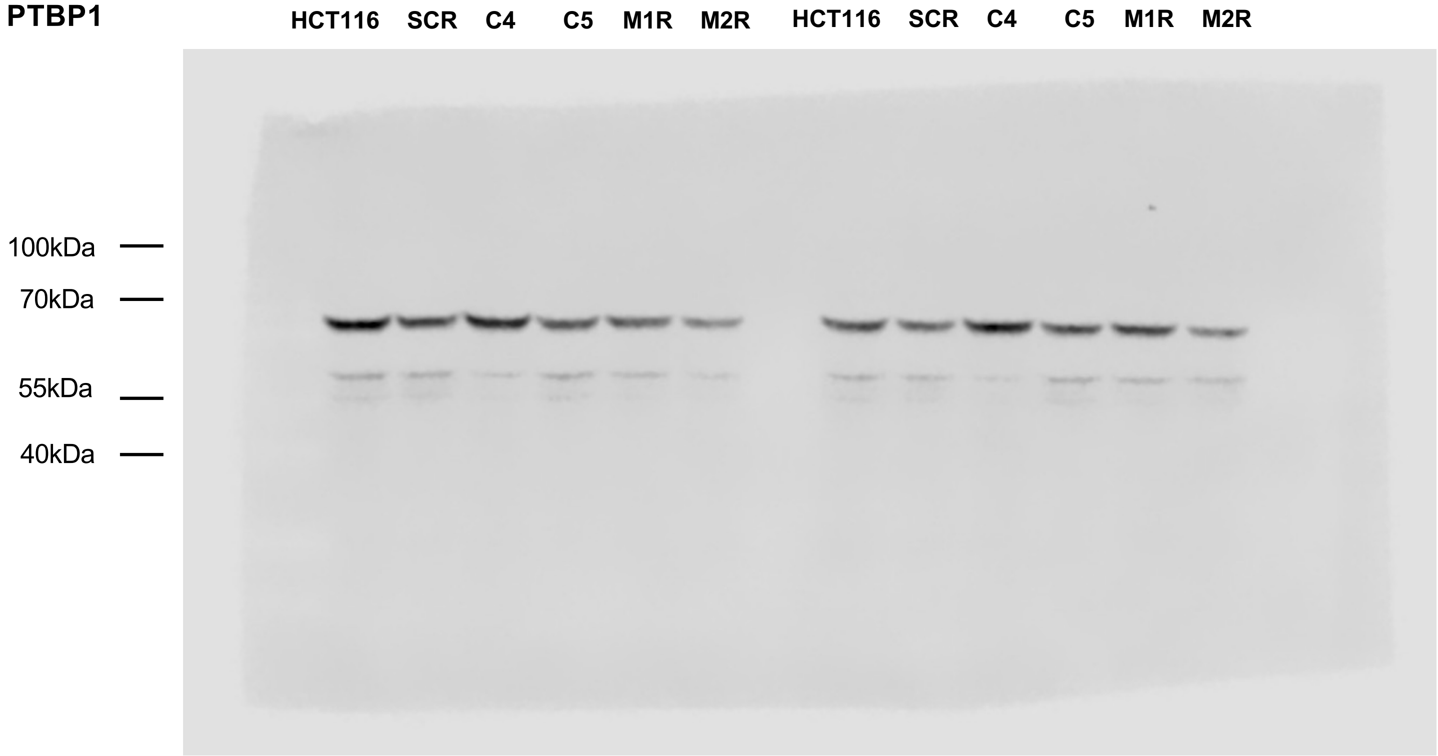

Supplemental Figure 1A

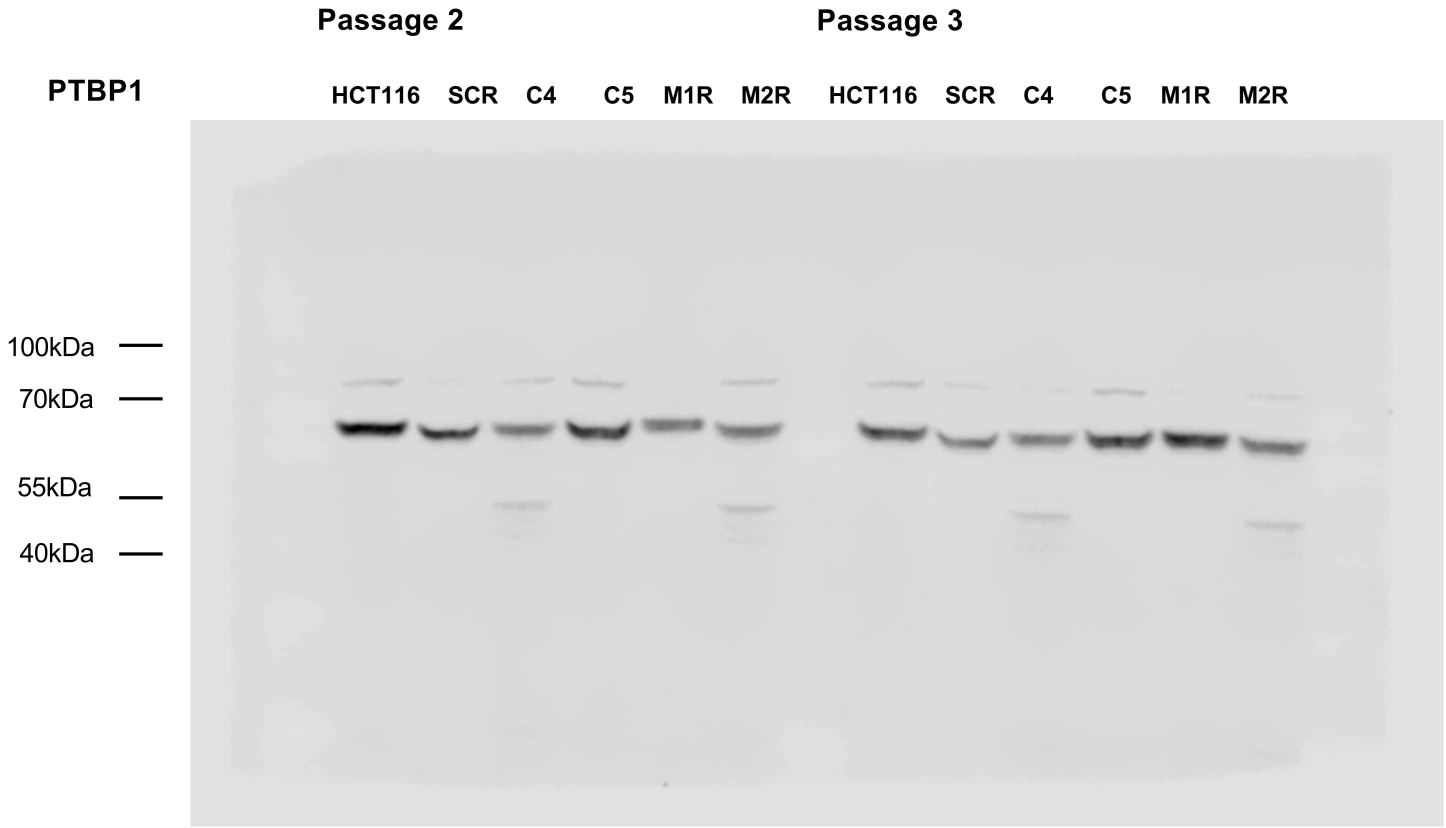

Supplemental Figure 1A

Passage 1

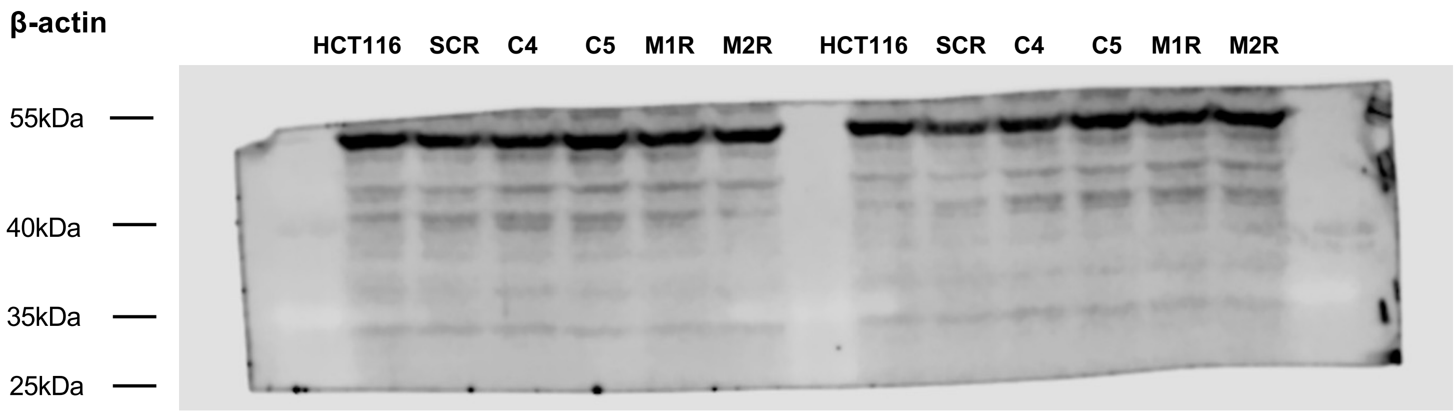

*Membrane was cut before primary Ab incubation.

Supplemental Figure 1A

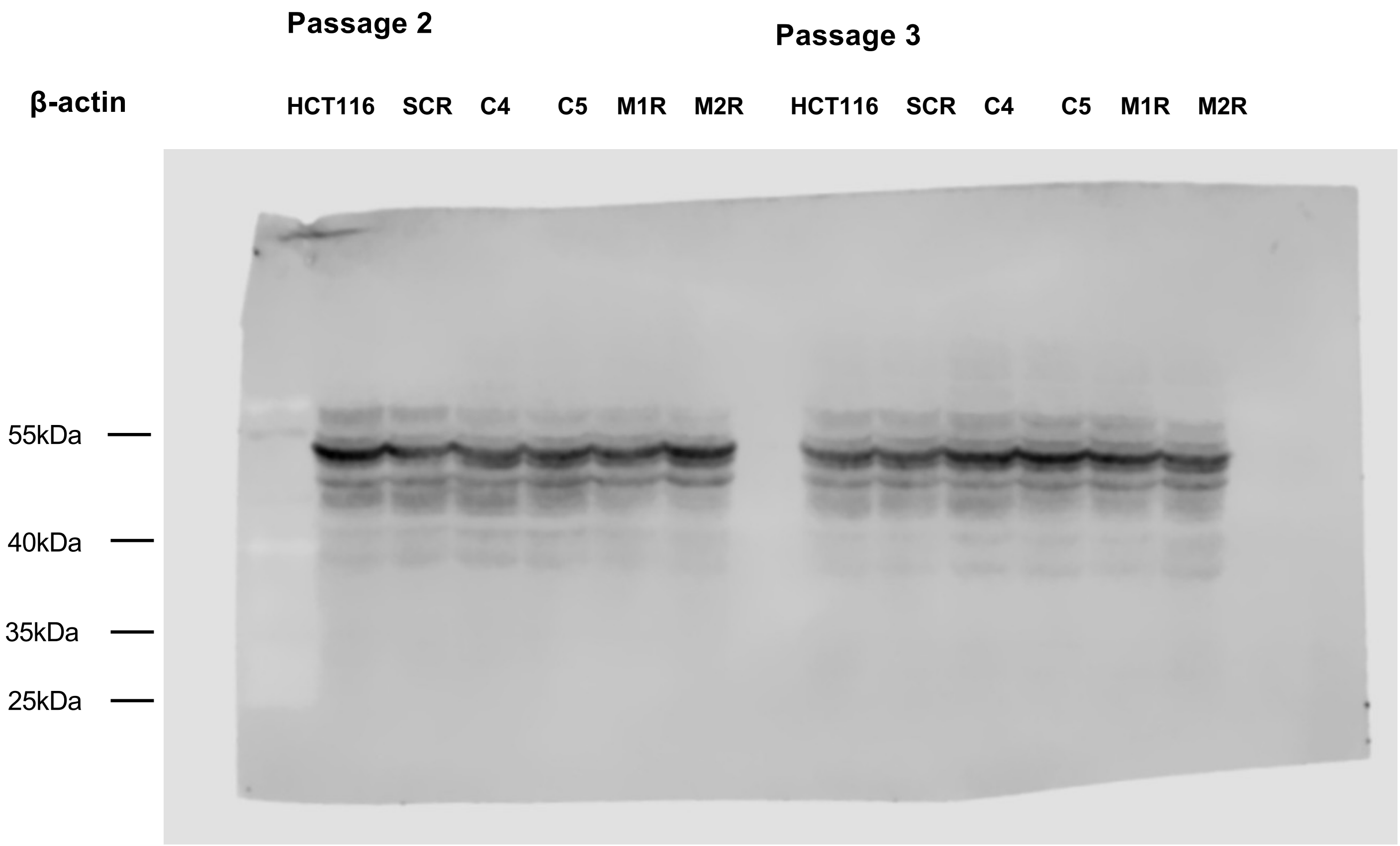

Supplemental Figure 2A

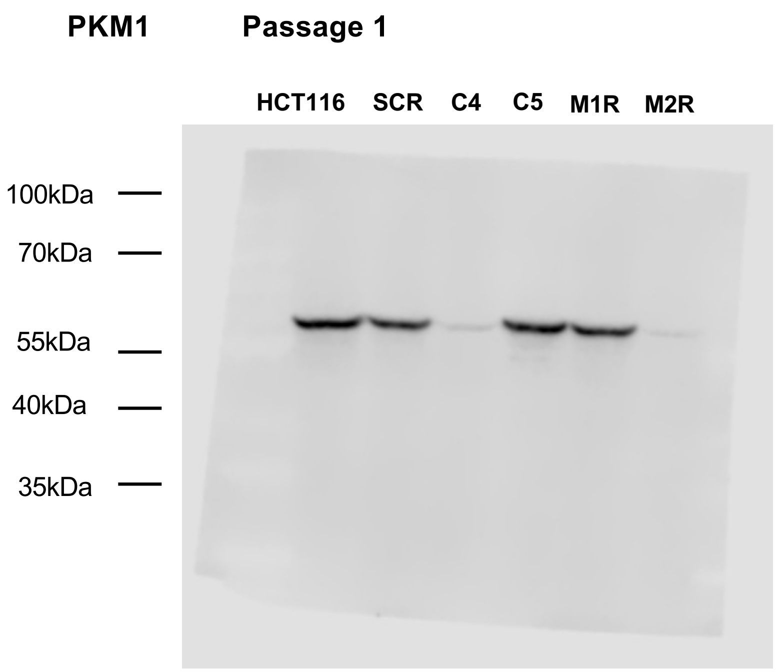

*Membrane was cut before primary Ab incubation.

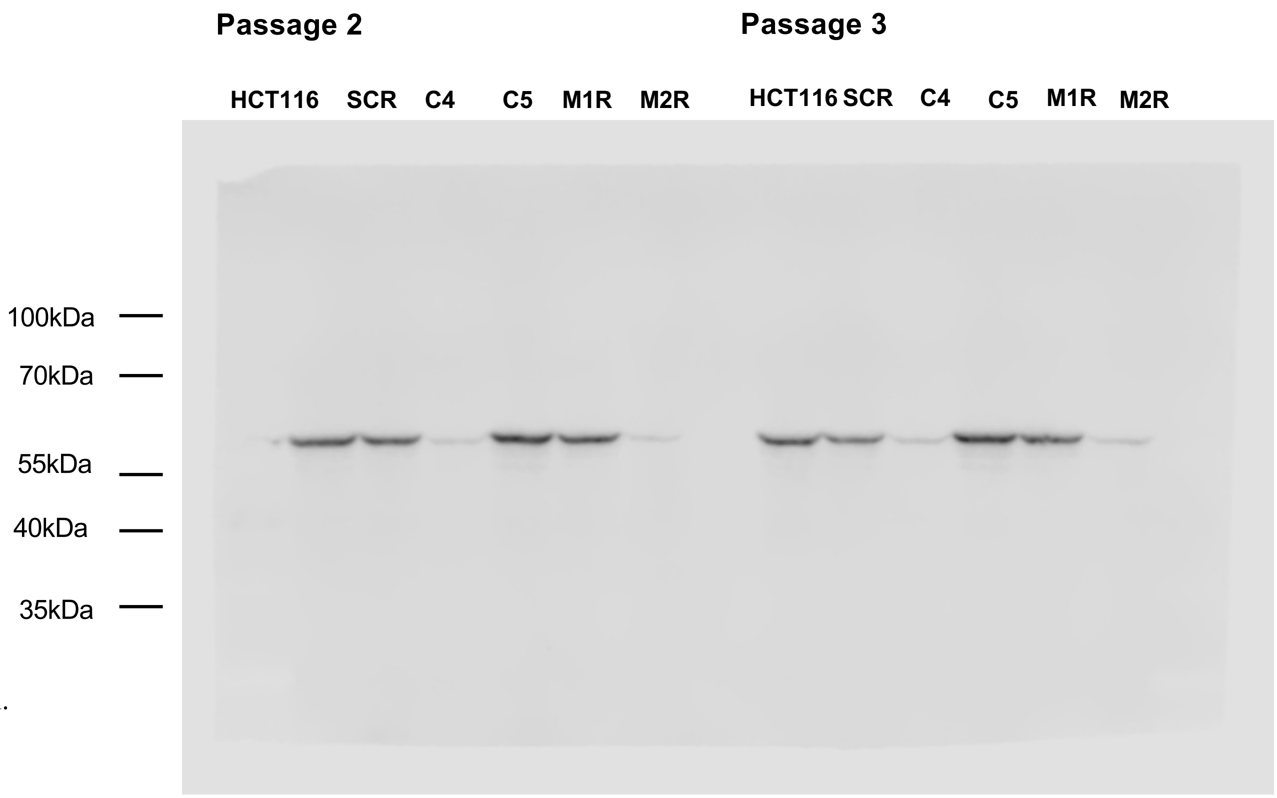

Supplemental Figure 2A

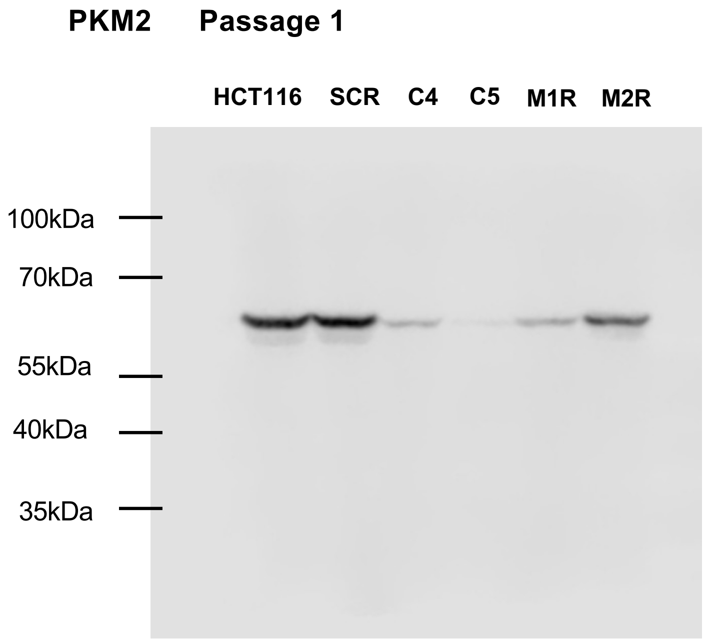

*Membrane was cut before primary Ab incubation.

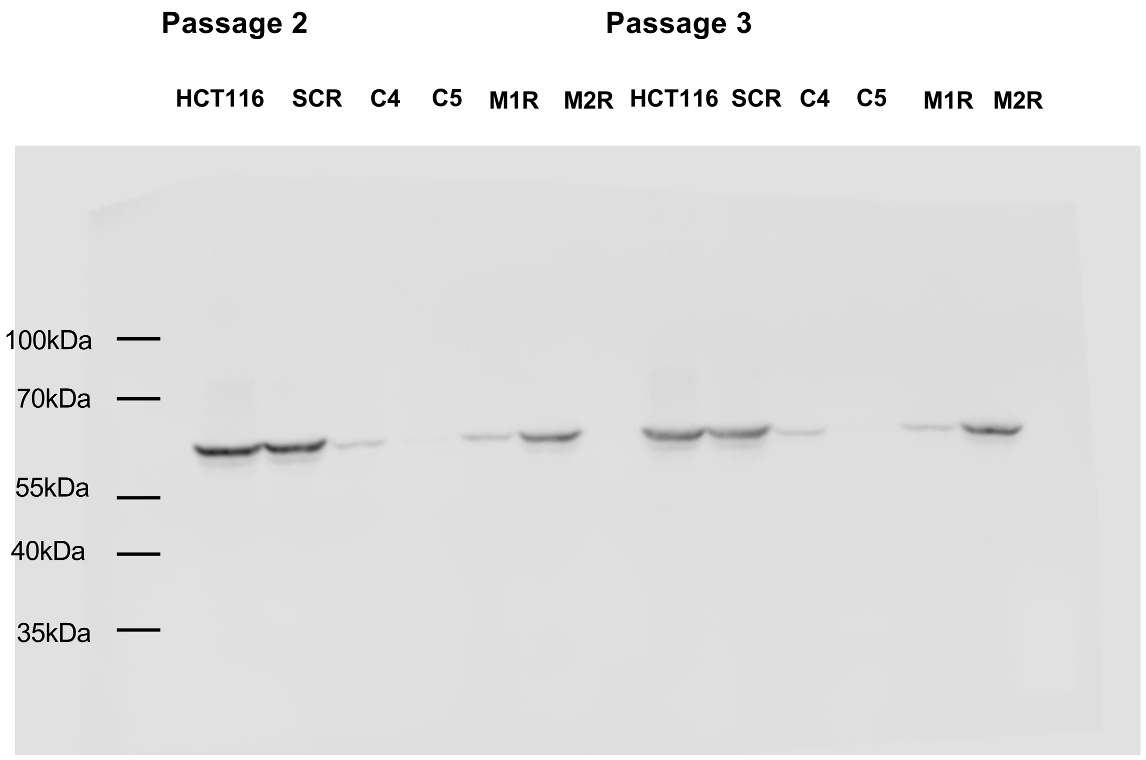

Supplemental Figure 2A

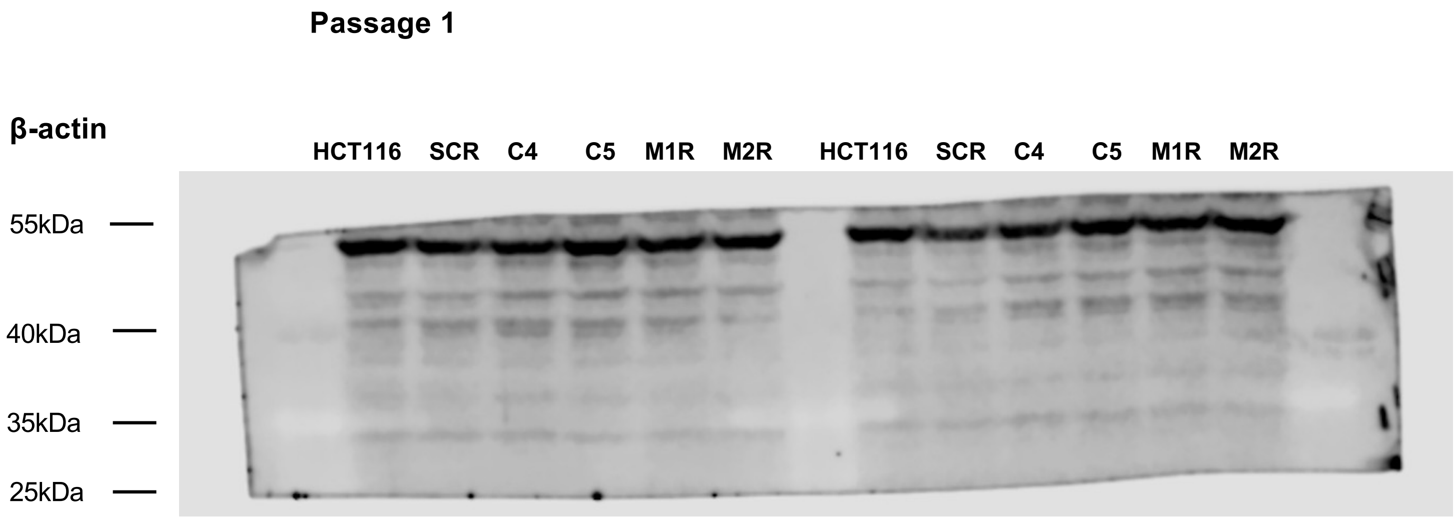

*Membrane was cut before primary Ab incubation.

Supplemental Figure 2A

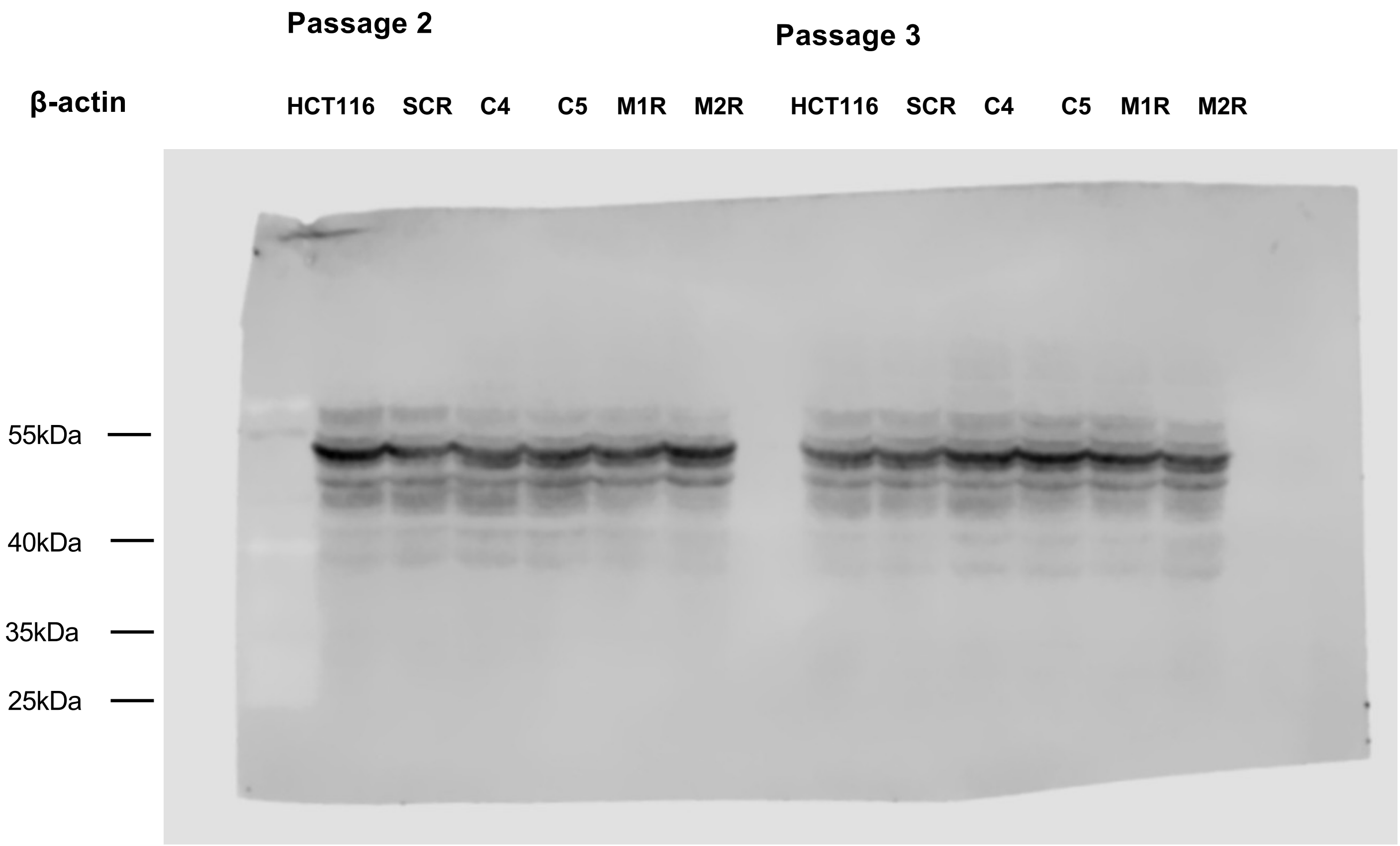

Supplemental Figure 3A

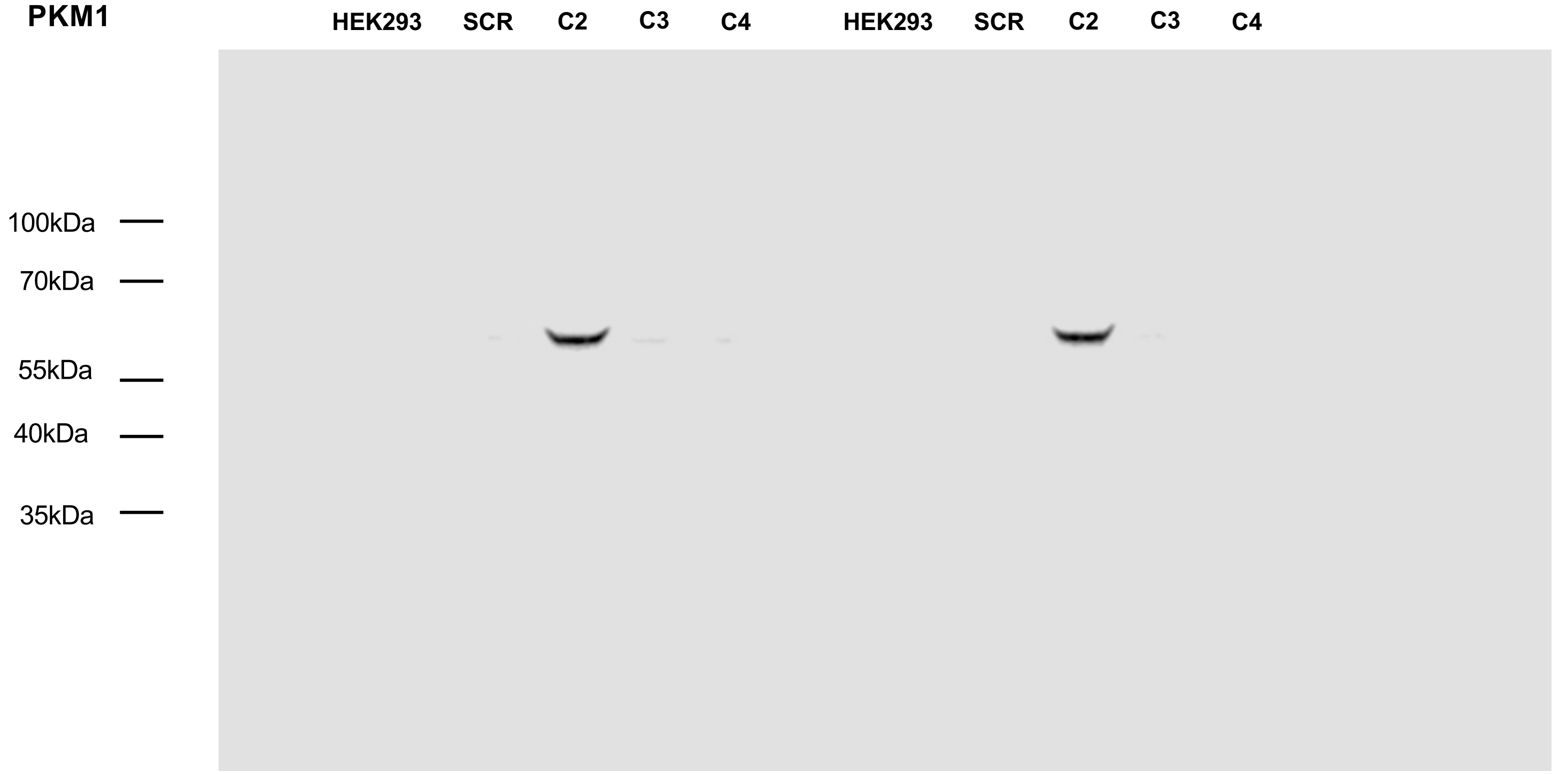

Supplemental Figure 3A

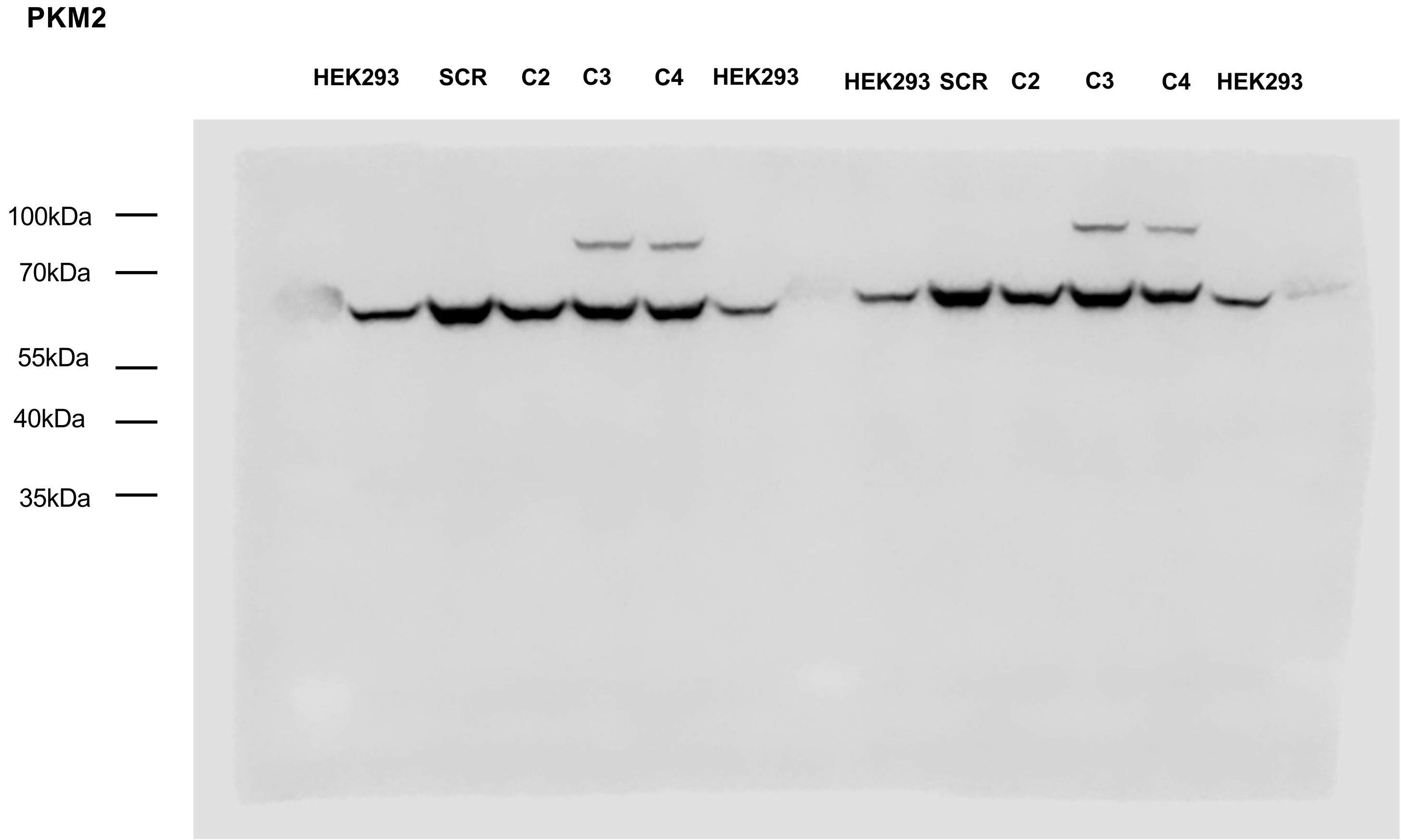

Supplemental Figure 3A

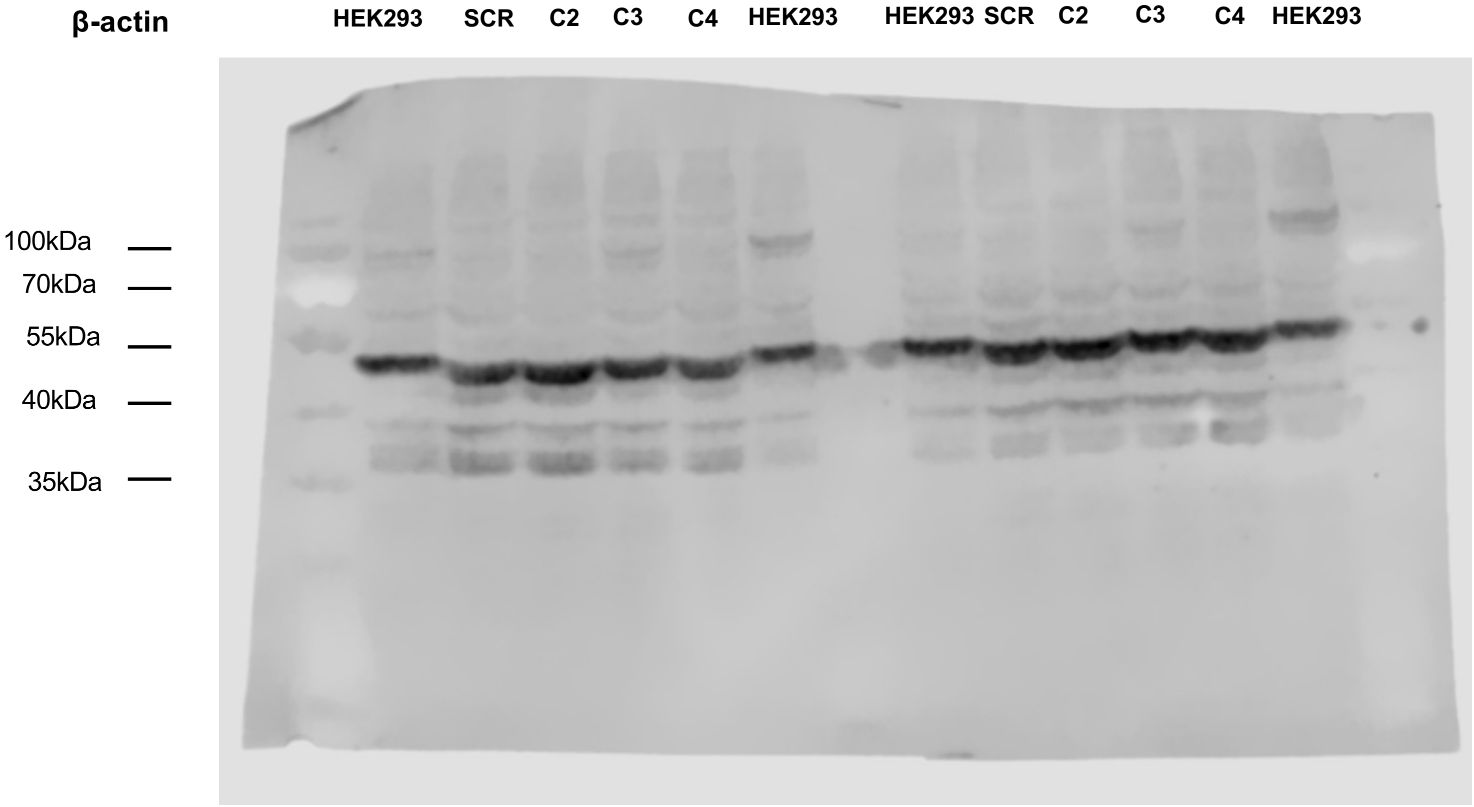

Supplemental Figure 5A

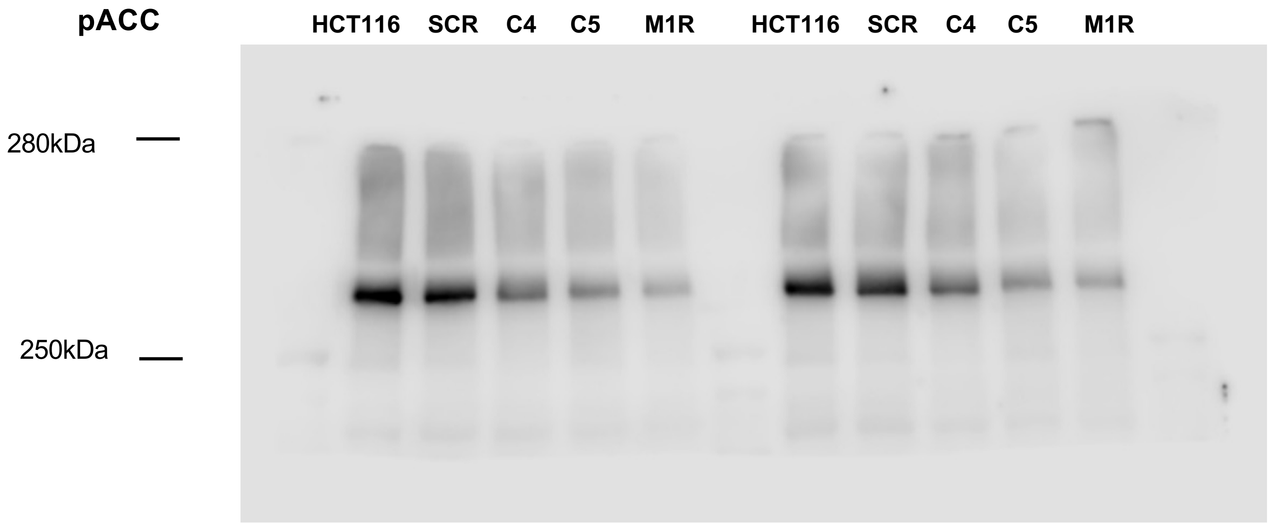

*Membrane was cut before primary Ab incubation.

Supplemental Figure 5A

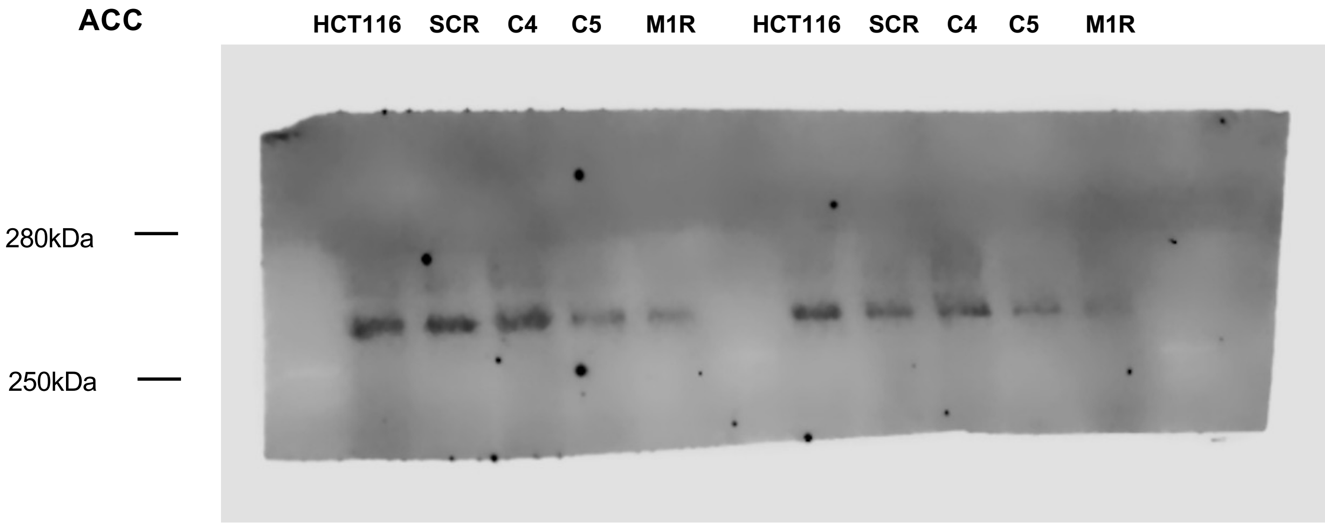

*Membrane was cut before primary Ab incubation.

Supplemental Figure 5A

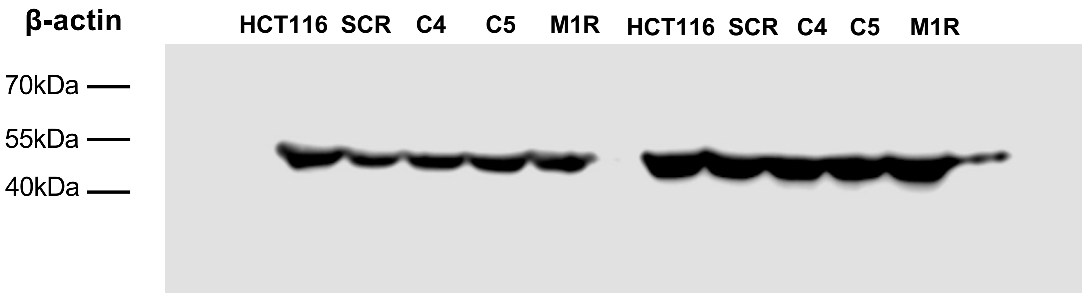

*Membrane was cut before primary Ab incubation.

Supplemental Figure 6A

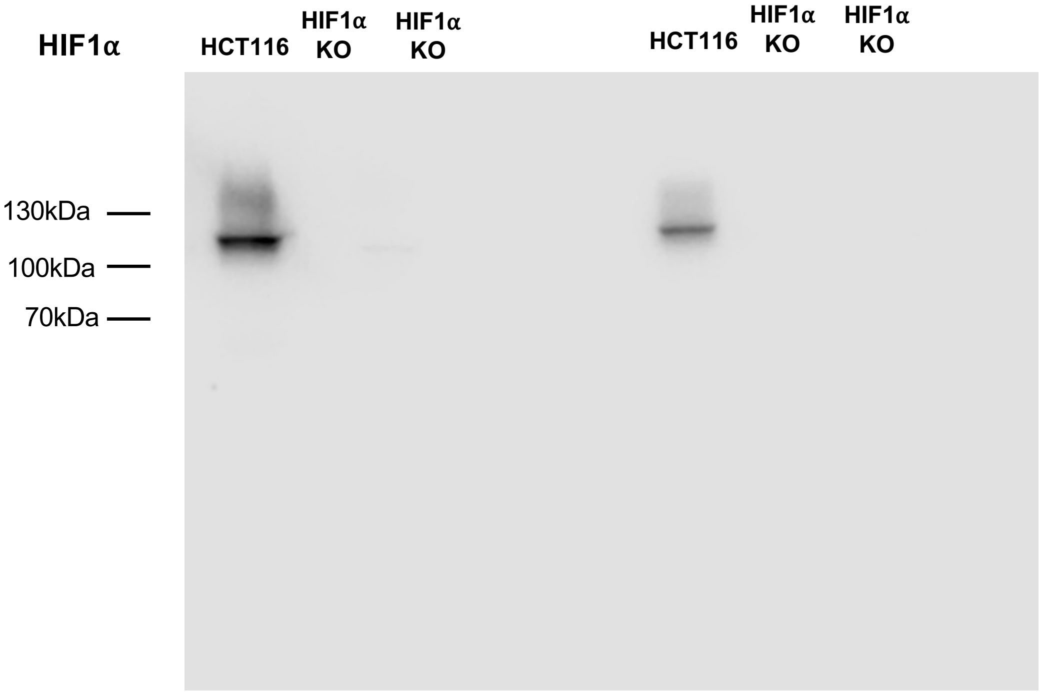

*Membrane was cut before primary Ab incubation.

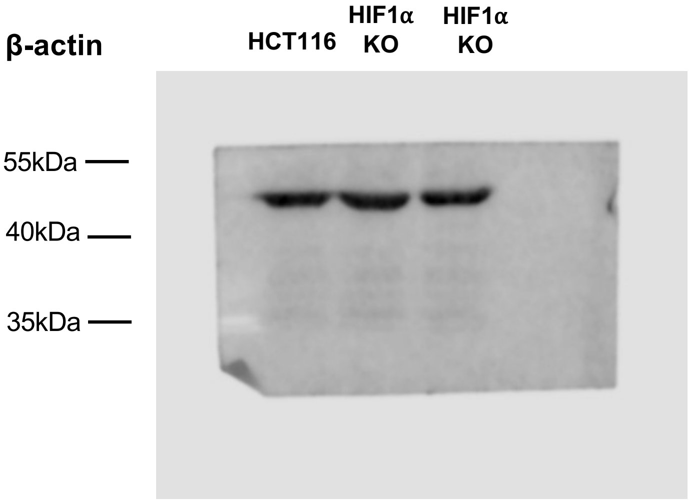

*Membrane was cut before primary Ab incubation.
